# Mapping job fitness and skill coherence into wages: an economic complexity analysis

**DOI:** 10.1038/s41598-024-61448-x

**Published:** 2024-05-23

**Authors:** Sabrina Aufiero, Giordano De Marzo, Angelica Sbardella, Andrea Zaccaria

**Affiliations:** 1 Dipartimento di Fisica, Università “Sapienza”, P.le A. Moro, 2, 00185 Rome, Italy; 2 Department of Computer Science, University College London, 66-72 Gower St, London, WC1E 6EA UK; 3 Centro Ricerche Enrico Fermi, Piazza del Viminale, 1, 00184 Rome, Italy; 4 Complexity Science Hub Vienna, Josefstaedter Strasse 39, 1080 Vienna, Austria; 5 Sapienza School for Advanced Studies, “Sapienza”, P.le A. Moro, 2, 00185 Rome, Italy; 6 Istituto dei Sistemi Complessi (ISC) - CNR, UoS Sapienza, P.le A. Moro, 2, 00185 Rome, Italy

**Keywords:** Statistical physics, thermodynamics and nonlinear dynamics, Computer science

## Abstract

Leveraging the discrete skill and knowledge worker requirements of each occupation provided by O*NET, our empirical approach employs network-based tools from the Economic Complexity framework to characterize the US occupational network. This approach provides insights into the interplay between wages and the complexity or relatedness of the skill sets within each occupation, complementing conventional human capital frameworks. Our empirical strategy is threefold. First, we construct the Job and Skill Progression Networks, where nodes represent jobs (skills) and a link between two jobs (skills) indicates statistically significant co-occurrence of skills required to carry out those two jobs, that can be useful tools to identify job-switching paths and skill complementarities Second, by harnessing the Fitness and Complexity algorithm, we define a data-driven skill-based complexity measure of jobs that positively maps, but with interesting deviations, into wages and in the bottom–up and broad abstract/manual and routine/non-routine job characterisations, however providing a continuous and endogenous metric to assess the degree of complexity of each occupational skill-set. Third, building on relatedness and corporate coherence metrics, we introduce a measure of each job’s skill coherence, that negatively maps into wages. Our findings may inform policymakers and employers on designing more effective labour market policies and training schemes, that, rather than fostering hyper-specialization, should favor the acquisition of complex and “uncoherent” skill sets, enabling workers to more easily move throughout the job and skill progression networks and make informed career choices decisions while unlocking higher wage opportunities.

## Introduction

Characterizing jobs in terms of human capital and understanding the relationship between skill requirements and wages are pivotal issues in the economic discourse, particularly for knowledge-based industries. This is increasingly crucial in the heated debate about the job characteristics complementary to automation^1,2^ or the occupational and sectoral shifts brought about by the sustainable transition^3–5^. Several contributions emphasise the importance of skill diversity and recombinant processes in knowledge generation^6–9^, however, treating human capital as uniform and skills as interchangeable falls short of acknowledging their multi-dimensional and diverse nature. A network-oriented and data-driven perspective may illuminate the disaggregated and interactive aspects of skill and knowledge recombination in each job, enhancing the information on the human capital content of occupations, while mitigating the inherent bias often associated with broad skill aggregation approaches^10^.

To this end, the present paper investigates the network structure and relatedness within the US occupational labor market. By relying on the discrete skill and knowledge worker requirements—which we will often refer to as skills for brevity—documented by O*NET for each SOC occupation, our empirical approach leverages various network-based tools from the Economic Complexity framework to characterize occupations based on their multi-dimensional human capital content. This approach provides insights into the interplay between wages and the complexity or relatedness of the skill sets within each occupation, complementing conventional human capital frameworks.

Our empirical strategy relies on the streams of literature on Economic Complexity (EC)^11,12^ and Evolutionary Economic Geography (EEG)^13^ and builds upon a growing body of literature that leverages the power of EC to gain insights into labor market dynamics^14–18^. The foundation of the literature on EC and EEG rests on the principles of evolutionary economics, particularly on the idea that the knowledge and capabilities embedded in local and national production systems are critical for fostering productive-technological diversification and economic growth. This view is encapsulated in the notion of ’relatedness,’ which posits that countries, regions, or cities are, if all other elements are unchanged, more likely to expand and diversify into industries and technologies that align with their existing specialization profiles and underlying resources^19,20^. While a unanimous operational definition of capabilities is still elusive^21^, there is consensus that the skills of workers, which can be regarded as actual “human capabilities”, serve as one of the foundational elements of production capabilities^22–24^. This holds true in both the knowledge economy and manufacturing, where tacit knowledge is embedded within production processes^23,25, 26^. From this vantage point, industries evolve along path-dependent trajectories influenced by the presence of human capabilities that are either related or complementary to the human capital characteristics demanded by firms. To measure occupational skill coherence, our approach is inspired by the concept of relatedness in the product space^20,27^, wherein export data is employed to assess product relatedness through co-exporting patterns in international trade networks, and the competitive exporters of a product are more likely to diversify into related goods, given their reliance on similar underlying productive capabilities.

The literature further emphasizes the complementarity of various types of capabilities in terms of knowledge spillovers. Patterns of related diversification are observed at both the national and local levels, as empirical co-occurrence networks reveal connections in specialization profiles not only in products but also in industries (see, e.g,^28,29^), technologies (see, e.g,^30–32^) or combinations of technologies, products, and scientific sectors^33–35^. Similarly to the case of productive capabilities, the empirical evidence highlights higher mobility of human capital between related industries, where firms frequently require similar skills^24,36, 37^. By examining skill-sharing in industry networks, where skill linkages across industries are established according to cross-firm labour flows, pioneering work by Neffke and Henning^24^ introduces the concept of skill relatedness, underscoring the key role of human capital in predicting firm diversification strategies^38,39^.

Our research shares common ground with other studies that have introduced the notion of *job space*—i.e. networks where occupations serve as nodes, and the edges’ weights are determined by different types of co-occurrences. For instance, occupational mobility has been explored relying on job spaces where the links depend on job switching probabilities. Focusing on the US, Del Rio-Chanona et al.^40^ found a positive relationship between job relatedness and occupational mobility; while Villareal^41^ identified a segmented job network structure. Axtell et al.^42^ and Lopez et al.^43^ used a firm-level labor flow network to propose a micro-foundation for frictional unemployment and to study the network interactions of job mobility and firm dynamics. Hartmann et al.^44^ analysed socio-economic segmentation based on job co-occurrences in Brazilian industries. Focusing on US cities, Muneepeerakul et al.^45^ investigated productivity dynamics through job co-location; while Farinha et al.^46^ studied the impact of job relatedness on entry/exit dynamics in the occupational structure using co-locations and co-occurrences of tasks or skills. From a complementary perspective, Alabdulkareem et al.^47^ and Anderson^10^ examined skill co-occurrences across occupations, exploring the *skill space*. The former authors linked skill topology to job polarization. The latter showed that workers with diverse skill portfolios tend to earn higher wages, highlighting the varying wage prospects associated with specific complementary skill sets. Finally, Gathmann and Schönberg^8^ explore human capital portability using a task-based approach. By defining a distance between occupations based on a task similarity, i.e. relatedness, measure, they show that task-specific skills are relevant drivers of wage growth.

As mentioned above, here, we study the relationship between jobs and skills using a network-based approach. To this aim, we construct two co-occurrence-based networks, the Job Progression Network (JPN) and the Skill Progression Network (SPN). In the Job Progression Network, two jobs are linked according to how often they require similar skills. In the Skill Progression Network, skills are linked when they are frequently (more often than randomly) required together in a significant number of jobs. While abstract occupations are quite spread across the JPN, two distinct communities representing technical and manual occupations are observed, and an even more profound separation between scientific-technical and humanities-communication communities is detected in the SPN, highlighting a profound labour market segmentation.

Projecting the SPN onto the occupational dimension, we then introduce *skill coherence* (or relatedness), which measures the similarity of skills within a job. Next, we apply the Economic Fitness and Complexity (EFC) algorithm^11^ to a bipartite network connecting jobs to their most important skill and knowledge requirements to assess *job fitness* (the overall complexity of the skills required by the job) and *skill complexity* (the sophistication and rarity of skills), providing a bottom–up, structural ranking of jobs and skills. The non-linear specification of the EFC approach to quantify complexity, which has proven to be more effective in maximizing the informative content in nested networks^48,49^, allows us to define a human capital metric, endogenous to the labour market and able to effectively quantify the degree of complexity of individual skills. The skill complexity metric permits us to study the different combinations of heterogeneous human capital attributes—more or less complex, more or less coherent—that characterize each occupation, leading to different degrees of a job’s fitness and wage prospects. Our findings suggest that job fitness is inversely related to skill coherence: complex jobs require diverse and less coherent skills, while low-fitness jobs rely on similar and compact skill sets. An overall positive relationship between job fitness and wages is detected, with notable exceptions at both the lower and the higher end of the wage scale, suggesting the prominence of the occupational dimension in determining wages in those cases^50,51^. Consequently, lower skill coherence is associated with higher wages, indicating that individuals with diverse and complex skill sets tend to earn more than specialists^10^. The connection between skill coherence and the broad and bottom-down abstract/manual or routinary/non-routinary job categorizations is also discussed, with non-routine job wages influenced by both skill coherence and job routinarity. Jobs with intermediate or high skill coherence, be they routine or non-routine, cannot escape a wage trap, only low-coherence non-routinary jobs and a few routinary jobs can access the entire range of wages.

By representing jobs and skills within a network framework, we can harness the analytical tools of Economic Complexity to gain a deeper and more detailed understanding of the endogenous and heterogenous characteristics of the US occupational structure and human capital requirements.

## Results

In this section, we provide some concise notes on the methodologies we employed, which are discussed more in detail in the Methods section, and we present the results of the application of different tools of the Economic Complexity methodology to the Skill-Job bipartite network obtained from the O*NET dataset (www.onetcenter.org). This database, fully described in the Methods section, enables us to define an adjacency matrix ${\textbf {M}}$, whose element $M_{js}$ is equal to 1 if the skill *s* is relevant for the job *j,* and zero otherwise. Sixty-eight different typologies of skills are reported, while the number of jobs depends on the aggregation level. In particular, we employ two occupational categories of the O*NET-SOC taxonomy (the O*NET elaboration of the Standard Occupational Classification commonly used in the United States): *Broad Occupations* comprising a total of 431 different jobs; *Minor Groups* including 95 job categories. The preliminary data processing approach and the construction of the Skill-Job network are detailed in the Methods Section below.

### The Skill and Job Progression Networks

The concepts of relatedness and coherence were originally introduced to study a firm-industry network by Teece et al.^52^, whose aim was to (i) reconstruct and quantify the possible overlap of capabilities between industries by counting co-occurrences of products across firms; (ii) use this measure of relatedness (or similarity) between industries to assess firms’ production coherence. As discussed in the introduction, in the EC framework similar approaches have been used to assess the relatedness of products by measuring their co-occurrences in the export baskets of countries^53^ through a network of products, referred to as the product space^20^. Note that in the EC literature the term *similarity* denotes the affinity between two nodes of the same set of the starting bipartite network (e.g., two products), while *relatedness* usually refers to nodes belonging to different sets (e.g., a country and a product)^54^. Here this distinction is not necessary and we employ interchangeably both terms. In order to quantify the relatedness between pairs of skills or jobs and to build the corresponding networks, we adopt the product progression network approach^27,55, 56^, that allows for statistical validation of the resulting network links. The rationale of our approach is that two skills are related if a significant number of jobs require both—for instance, our findings indicate that *Mechanical skills* and *Equipment maintenance*, or *Troubleshooting* and *Quality control analysis* co-occur more often than random. Analogously, two jobs are related if they share a significant number of their skill requirements. By following the methodology proposed by Zaccaria et al.^27^, to take into account the nested structure of the adjacency matrix^57^ we normalise these simple co-occurrences of skills in jobs by job diversification $d_j=\sum _s M_{js}$ and skill ubiquity $u_s=\sum _j M_{js}$. In practice, we compute:

1 $$\begin{aligned} B^{Jobs}_{jj'}=\frac{1}{\max \left( d_j,d_{j'} \right) }\sum _{s}\frac{M_{js}M_{j's}}{u_s}. \end{aligned}$$

as a measure of the relatedness between job *j* and job $j'$, and:

2 $$\begin{aligned} B^{Skills}_{ss'}=\frac{1}{\max (u_s,u_{s'})}\sum _{j}\frac{M_{js}M_{js'}}{d_j} \end{aligned}$$

as a measure of the relatedness between skill *s* and skill $s'$.

The resulting networks, defined by the adjacency matrices ${\textbf {B}}^{Skills}$ and ${\textbf {B}}^{Jobs}$, are almost fully connected (almost all possible edges are present) and may contain spurious links, as observed in the country-product case^58^. As a consequence, to ensure that only meaningful connections between jobs and skills are considered and spurious links are filtered out, a statistical validation procedure is required. In particular, we rely on the Bipartite Configuration Model proposed by Saracco et al.^59^ and based on node heterogeneity. As in the normalization procedure, the statistical validation aims to filter links present only because a co-occurring job is highly diversified, or a co-occurring skill is highly ubiquitous. Further details on the validation strategy are provided in the Methods Section.

**Figure 1:**
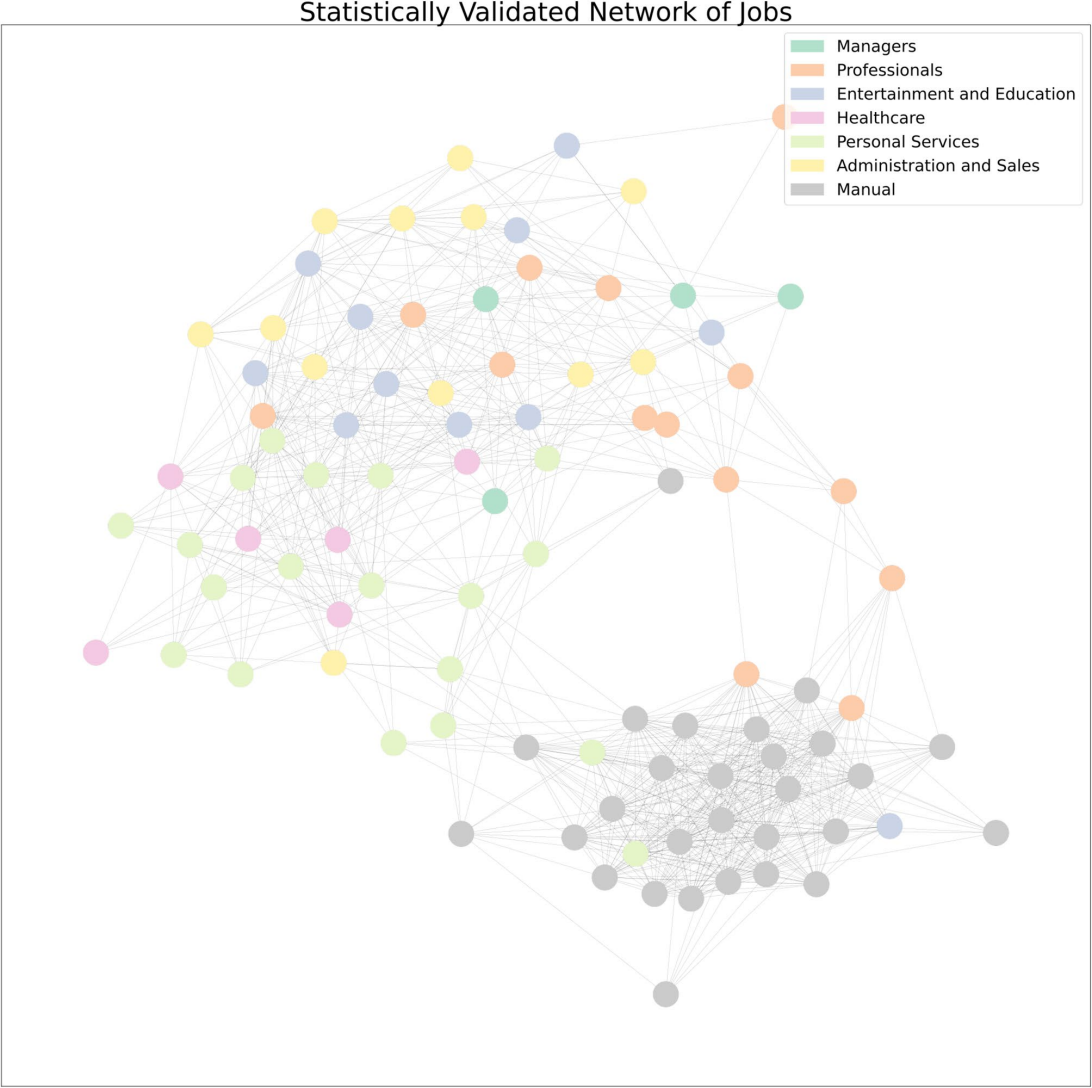
The Job Progression Network. Statistically validated network of 95 jobs. Each node represents an occupation and the links are given by the normalised overlap of skills. A technical/manual job community emerges, while abstract occupations appear to be more spread throughout the rest of the network.

We first use this strategy to construct the Job Progression Network (JPN) depicted in Fig. 1, where nodes represent jobs and links are given by normalized and statistically validated co-occurrences of skills. Intuitively, in this representation jobs that share a high number of skills, i.e. they rely upon similar *human capabilities*, are close to each other in the network. Therefore, the observation of the network allows us to trace the most feasible trajectories for moving to a new occupation on the basis of their shared skills. To illustrate, jobs sharing similar inputs include “Supervisors of food preparation and serving” and “Cooks and food preparation”, or “Agricultural” and “Fishing and hunting”.

As can be appreciated from the colour distribution of the nodes in Fig. 1, the structure of the JPN portraying job relatedness as measured by co-occurrences of skills in occupations does not display a one-to-one correspondence with the O*NET-SOC hierarchical job taxonomy. In fact, while colours in Fig.  1 correspond to seven macro-occupational categories obtained aggregating O*NET-SOC Major Groups (Managers, Professionals, Entertainment and Education, Healthcare, Personal Service, Administration and Sales, and Manual Occupations), two distinct communities across different macro-occupations can be observed in the plot. The first community in the bottom right corner covers manual occupations, and the second in the upper left corner densely connects different high- and low-skill jobs, ranging from managerial and professional to education, healthcare, and personal services, regardless of their position in the classification. This community structure is also confirmed by means of community detection algorithms, as detailed in the SI. In particular, the detected communities appear to be stable across three different algorithms we exploited. A more detailed description of the network and the occupations contained in the two communities can be found in the SI.

The structure of the network points out the different and complementary information provided by job relatedness: while the O*NET-SOC classification reflects the final occupational requirements and worker attributes, our approach is focused more on *how* the skill compositions of two jobs are related to each other: in tune with the economic complexity literature, here we interpret skills are *human capabilities*, the underlying and interconnected building blocks necessary to perform a job. This network of jobs may thus constitute a road-map to plan retraining and investment strategies to overcome skill obsolescence, help workers to more easily move to a coherent new occupation, and foster on-the-job trainings aimed at developing cross-cutting non-task-specific competencies, in particular abstract (cognitive and digital) skills with the aim of diminishing the gap between the two significantly disconnected job communities.

**Figure 2:**
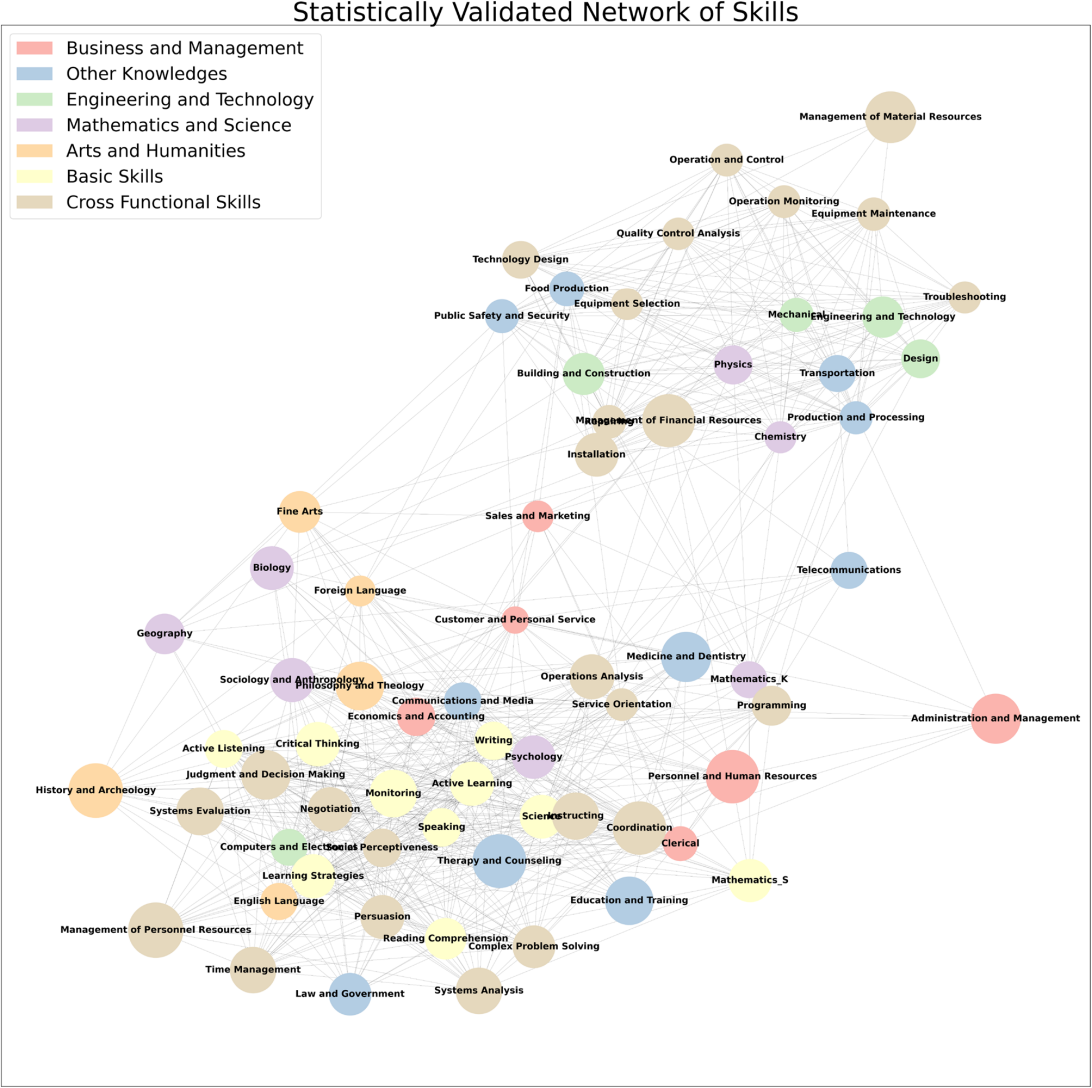
Skill Progression Network. Statistically validated network of 68 skills. Each node represents a skill and the links are given by normalized co-occurrences of skills across jobs. Scientific and technical skills are mainly found in the upper community and are mainly independent from humanities and communication skills, found in the lower community.

Next, Fig. 2 represents the Skill Progression Network (SPN), where the colours of the nodes reflect the O*NET skill and knowledge classifications, and two skills are close if a significant number of jobs require them both. For instance, examples of highly related skills include: *“Mechanical”* and *“Equipment maintenance”*; *“Equipment selection”* and *“Operation monitoring”*; or *“Troubleshooting”* and *Quality control analysis*. It is important to notice that in constructing the network of skills we find a strong dependence of the relatedness between skills (${\textbf {B}}^{Skills}$) from the job classification. At a low aggregation level (Minor Groups with 95 occupational categories), a large number of unrelated skills co-occur just because the job definition is very wide. This leads to links between skills that are unlikely related, such as *“Medicine and dentistry”* and *“Law and government”*, or *“Food production”* and *“Economics and accounting”*. To avoid this occurrence and remove such source of noise, when computing ${\textbf {B}}^{Skills}$, we use the most detailed job classification (Detailed Occupations with 873 occupational categories). This is analogous to what is observed for product relatedness: product co-occurrences at the country level show a number of spurious correlations which can be removed if firm level data is used^58^. In both cases, a better computation of the relatedness of one layer (skills or products) can be obtained by using a more detailed aggregation of the other layer (Detailed Occupations or firms′s products).

Also in the case of the SPN in Fig. 2 we detect two distinct communities. The first community is placed in the top right corner of the network and is associated with industrial production processes being mainly composed of cross-functional technical (brown), scientific (purple), engineering, and technical (green) skills—the latter category is almost exclusively represented in this community, with only the exception of *“Computer and electronics”*. The second community can be found in the lower-left corner of the plot: more populated than the first, it densely connects an ensemble of more abstract skills. It covers all basic (yellow) and art and humanities (orange) skills present in O*NET, while it also displays a fair share of cross-functional and scientific skills and health services-related skills. Business and management skills (red)—e.g. *“Sales and marketing”*, *“Administration and management”*, and *“Customer care and personal service”*—together with *“Telecommunications”* appear to have a tight web of links with both communities, thus acting as the connecting thread between the two groups of skills. We report in the SI a more detailed description of the network and the skills found in the two communities. This non-trivial network structure highlights the underlying differences in the skills classified in the same category, emphasising complementarity also between sets of competencies apparently unrelated. Also in this case we applied a community detection algorithm to validate the communities we visually identified. As detailed in the SI, in this case, different algorithms tend to find different communities, but the technical skill community is always present, confirming the robustness of such a structure. In conclusion, our approach thus is not only able to assess the presence of a skill-based relatedness between jobs but may also provide a quantitative measure of the relatedness degree between otherwise undetectable links of skills, possibly opening up new developments and applications in designing training or retraining strategies.

### Jobs complexity, coherence, and wages

In this section, we discuss an algorithmic assessment of the complexity of jobs based on their skill content. We refer to this metric as *job fitness* and we compute it by applying to the O*NET occupational data the Economic Fitness and Complexity (EFC) algorithm, originally introduced by Tacchella et al.^11^ for assessing the complexity of national economic structures in the international trade country-product network. The rationale of this version of the EFC algorithm is that a job requiring a diversified set of skills, comprising both sophisticated—i.e., more *complex*—and basic skills, must display higher fitness than a job requiring only a few basic skills.

Therefore, we use the EFC algorithm’s two coupled iterative equations to compute the *job fitness* $F_j$ of job *j*, and the *skill complexity* $Q_s$ of skill *s*. According to the first equation, $F_{j}$ is defined as the sum of the complexity of the skill required to perform *j*, thus a job displays high fitness if it is associated with many complex skills. The equation that defines the complexity of skills is, instead, not linear. In fact, since high-fitness jobs require both complex and basic skills, to determine whether a skill is complex, the key information is conveyed by the skill content of low-fitness jobs. If a job presents low skill requirements, we expect all of the associated skills to be basic. Consequently, if a low-fitness job requires a given skill, this skill will feature low complexity. More details about the mathematical construction and the convergence properties of the algorithm are provided in the Methods section. In formulas, the EFC algorithm is defined as follows:

3 $$\begin{aligned} F_{j}^{(n)}= & {} \sum _{s} M_{j s} Q_{s}^{(n-1)} \nonumber \\ Q_{s}^{(n)}= & {} \dfrac{1}{\sum _{j} M_{j s} \dfrac{1}{F_{j}^{(n-1)}}} \end{aligned}$$

where $F_j$ is the fitness of job *j*, $Q_s$ is the complexity of skill *s*, and *n* is the iteration number of the algorithm. The fixed point of this map does not depend on the initial conditions^60^ and has non-trivial convergence properties^61^.

**Figure 3:**
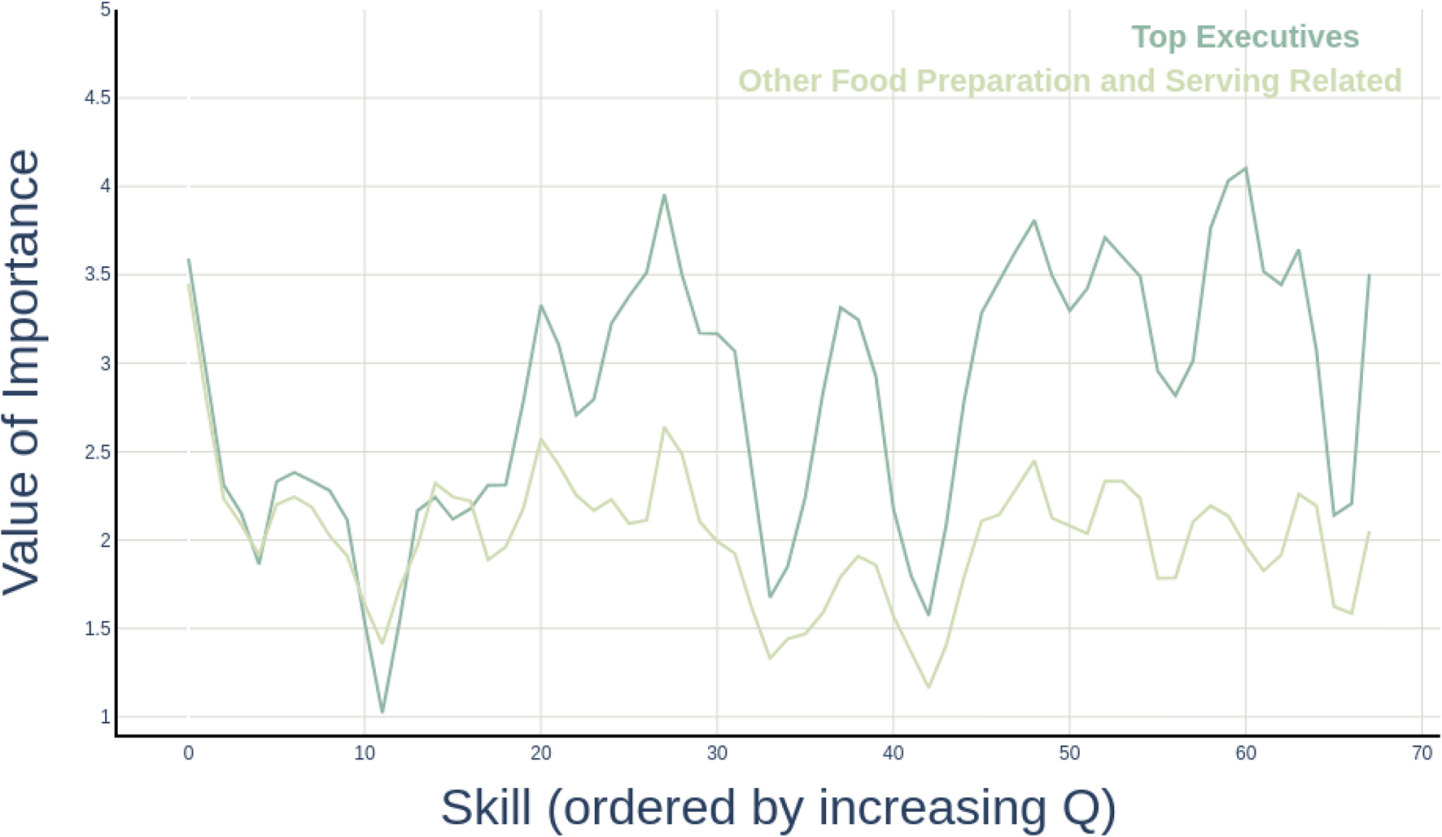
Skill spectroscopy of jobs. Examples of one high- and one low-fitness job are provided. The low fitness job, “Food preparation and serving” in light green, requires a diverse set of skills ranging across skill complexity levels, however, all the skills associated to it (especially the complex ones) display a low level of importance for the occupation. By contrast, the highest fitness job, “Top Executives” in dark green, not only relies on a larger set of complex skills nut they also display high values of importance.

The job fitness rankings resulting from this formulation of the EFC algorithm confirm our intuition about the high number of sophisticated skills required in high-fitness occupations. Our measures of job fitness and skill complexity are fully available in the SI. Among the top fitness jobs, we find “Management and Top Executives”, as well as “Supervisors of Repair and Protective Service Occupations”, but also “Architects” and “Life Scientists”. By contrast, low-fitness jobs include “Food Preparation and Serving Related Workers”, “Entertainment Attendants”, “Financial Clerks”, “Personal Appearance, and Administrative Support workers”. By following the graphical representation proposed by Zaccaria et al.^62^ for the *product spectroscopy of countries*, where each country’s exported goods are plotted against their respective complexity, to visualise the skill fingerprints of a job it is possible to build the *skill spectroscopy of jobs*. This kind of graphic visualisation is analogous to spectroscopy analysis in materials science, where each material is uniquely identified by the spectrum of the electromagnetic radiation it emits^63^. In Fig. 3 we provide an example for the skill spectroscopy of one high-fitness job, “Top Executives” in dark green, and of a low-fitness job, “Food preparation and serving” in light green. On the horizontal axis, skills are sorted by increasing complexity—where *“Quality Control Analysis”*, *“Customer and Personal Service”*, *“Equipment Selection”* are examples of low complexity skills; while *“Therapy and Counseling”*, *“Coordination”*, *“Management of Financial Resources”* of high complexity skills. On the vertical axis, we report the skill *importance* for each specific job provided by O*NET, with importance equal to one corresponding to the lowest level of skill mastering—see the Methods section for more details on the data-set. In our example, the importance of each low-complexity skill is very similar for the two selected occupations, as can be observed by the concordant trends followed by the two curves in the left portion of Fig. 3. Moving to intermediate and especially high complexity on the x-axis, we observe a growing discrepancy between the curves in the plot, with complex skills appearing as the main driver of the different positions occupied by the two jobs in the fitness ranking.

**Figure 4:**
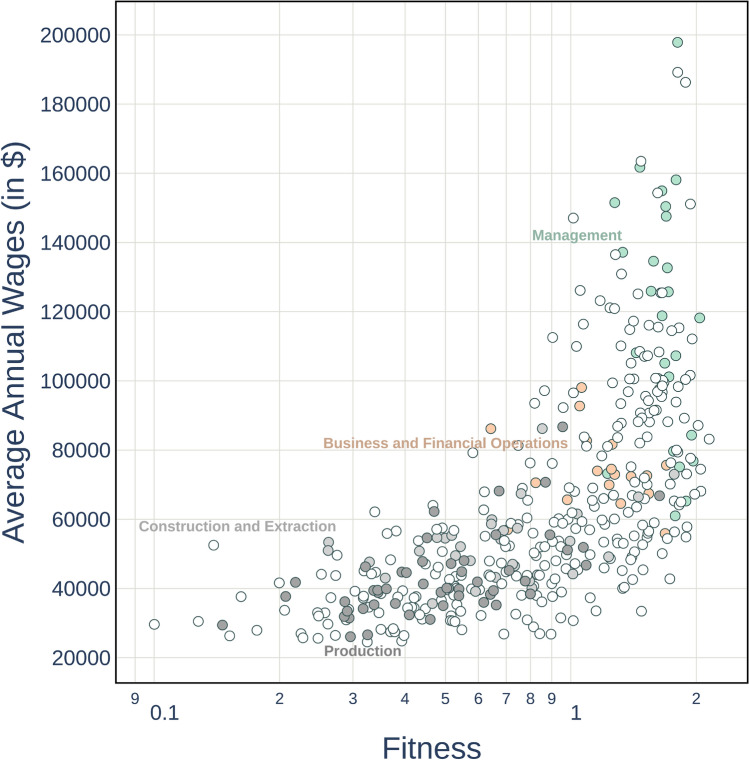
Relationship between job fitness and average wage; where as jobs the O*NET Broad Occupations are considered. The relationship appears to be broadly positive, with a high share of low-fitness jobs confined in the low-wage area. Many high-fitness occupations are however rewarded by heterogeneous wages, independently of their skill content.

Going beyond the broad, top–down dichotomy between abstract versus manual, or routinary versus non-routinary occupations, our fine-grained and bottom–up approach might offer new insights on the skill-wage relationship. Therefore, in Fig. 4 we check how the fitness of jobs, computed as a skill complexity-weighted diversification, is associated with wage levels—where each scatter plot point represents a job belonging to an O*NET Broad Occupation category.

From the plot it is possible to observe a strong but nonlinear correlation between fitness and wages, suggesting that the labour market does not always reward occupations with a diversified and complex skill content. The relationship appears to be broadly positive, with more complex skill requirements corresponding to higher average wages, especially for low and intermediate fitness levels. However, noteworthy deviations from such a positive trend are detectable, especially at the two extremes of the wage distribution. First, a share, albeit small, of low-fitness jobs is confined in the low-wage area, with jobs in production or construction (respectively in dark and light grey in the plot) featuring similarly low annual average wages despite showing a heterogeneous distribution of fitness—with, e.g., “Laundry and Dry Cleaning Workers” earning 26,600 US dollars per year, and “Power Plant Operators, Distributors, and Dispatchers” earning 86,720 US dollars per year. Second, for the highest fitness occupations we detect a much stronger decoupling between a job’s complexity skill content and wages, as can be observed by the vertical distribution of the points on the right portion of the plot corresponding to jobs with similar fitness levels and a wide range of high wages, suggesting the underlying presence of relatively skill-independent wage setting mechanisms for very high wages. This high wage premium is particularly evident in the plot for managerial occupations (in green): managers often share similar skill profiles, and thus fitness, with the bulk of business and financial occupations—the majority of which is placed in the 60,000–80,000 US dollars per year region—however not only they exhibit higher wages but also higher within-occupation wage differentials at similar job fitness levels—with the extremal cases of “Food service Managers earning” 61,000 US dollars per year, and “Engineering Managers” earning 15,8100 US dollars per year. In line with the studies of top income earners^50^, especially for top executives or in financial occupations^64^, the ratio of the highest paid manager’s salary (“Chief Executives”, 19,7840 US dollars per year) to the lowest paid (“Food Service Managers”) is 3.2; the ratio of the highest paid manager’s salary to the lowest paid occupation in general (“Fast Food and Counter Workers”, 24,540 US dollars per year) is 8.06. Wages do not always reflect an occupation′s intrinsic value in terms of skills, they are not simply the outcome of well-functioning competitive markets rewarding skills based on marginal differences: at the very bottom of the pay scale a sort of wage trap emerges, apparently driven more by the occupational category than its skill requirements; in contrast, when focusing on high-skilled occupations higher wage is accumulated at the very top of the pay scale, regardless the diversification or complexity of the job’s skill set.

**Figure 5:**
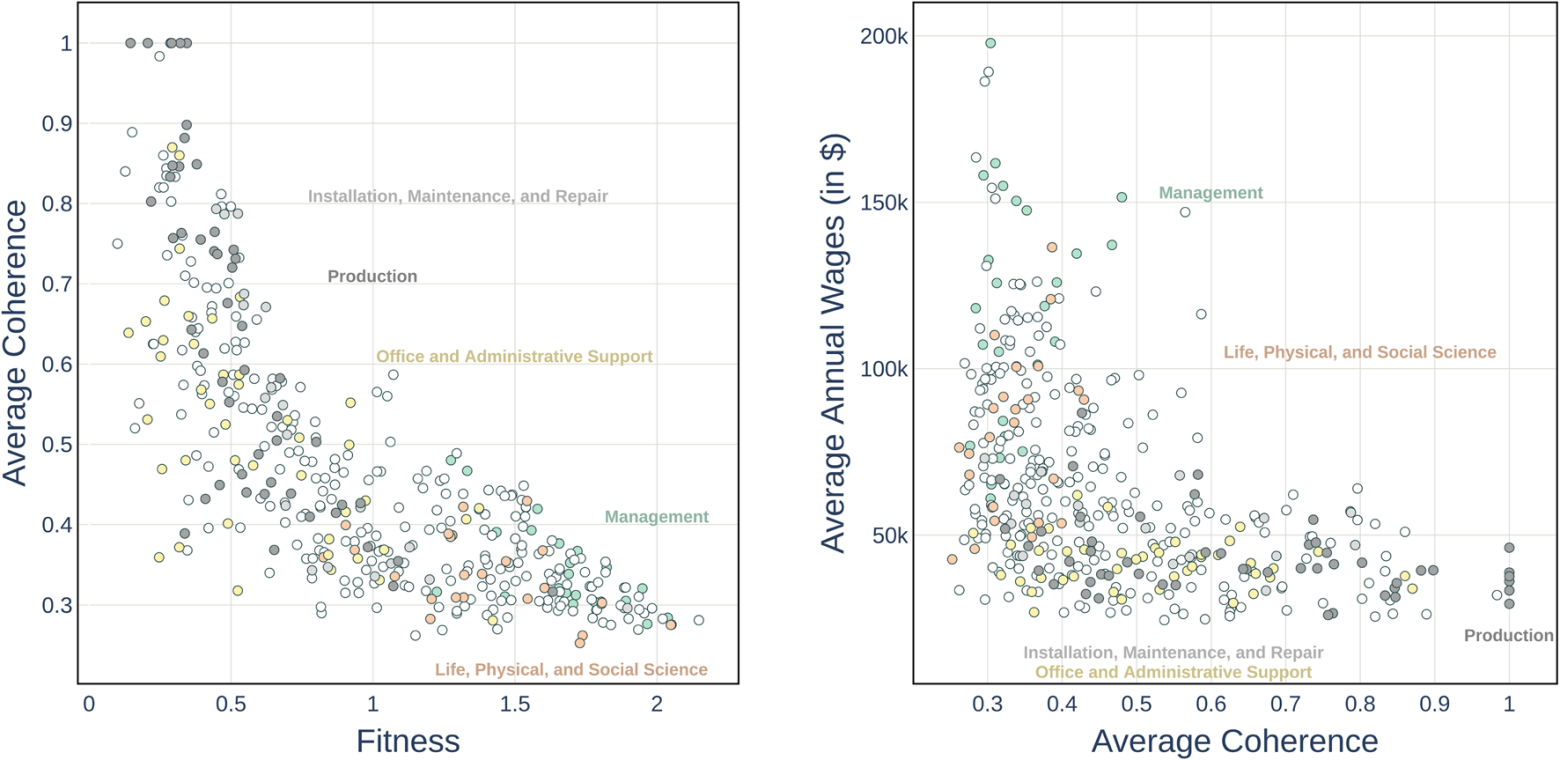
Left panel: Job Fitness versus Job Average Coherence (AC); right panel: AC versus occupational annual average Wages. AC and Job Fitness are negatively correlated. Occupations requiring very similar skills show low wage levels; however, having a low AC is not a sufficient condition for unlocking a high wage.

As detailed above and in the Methods Section, Job Fitness measures the degree of sophistication of each occupation′s skill requirements, giving also importance to the skill mix diversification. However, such a measure does not capture the possible role played by the degree of coherence (or lack thereof) of each job's skill requirements, i.e. their *relatedness*, which may potentially provide additional explanatory power in studying the relationship with job fitness and wages. To this aim, we exploit the relatedness measure ${\textbf {B}}^{Skills}$ introduced in the previous section to measure the degree of skill coherence in each occupation, that is, the average relatedness between the skills it requires. We denominate this quantity job Average Coherence (AC) and compute it by averaging the relatedness matrix $\textbf{B}^{Skills}$ over each pair of skills ${s,s'}$ required by job *j* according to O*NET, i.e.:

4 $$\begin{aligned} \text {AC}_{j} =\frac{\sum _{s s'} {M}_{js} {M}_{js'}B_{ss'}}{\sum _{s s'} {M}_{js} {M}_{js'}} \text{. } \end{aligned}$$

A job with a high value of AC is *coherent*, i.e. it requires skills co-occurring in the same occupations more significantly than random. By contrast, if the required skills are not often associated, the value of AC will be low. Note that this quantity is, by construction, independent from the total number of skills and thus provides complementary information with respect to that provided by fitness. In the two panels of Fig. 5, for each occupation, we show the relationship between job AC and fitness (left) and wages (right), respectively. The Supplementary Information includes the same figures in an interactive HTML format, allowing for a more dynamic exploration of the plots. From the left panel, it is possible to observe that AC provides a piece of information mainly negatively correlated to fitness: complex jobs feature more heterogeneous skill requirements, while low-fitness jobs present a highly coherent skill mix. More in detail, a large share of highly coherent jobs show low fitness and are characterized by similar skills, consistently with the technical community observed in Fig. 1 – for instance operators in the “Installation, Maintenance and Repair”, “Production”, or machine operators“Sewing”, “Woodworking”, and “Molding” occupational categories. Clerical jobs generally display low fitness values but a relatively high degree of coherence, suggesting that their distinctive skills, albeit relatively simple, are quite similar. Finally, “Management” occupations share with “Life, physical, and social scientists” a high value of fitness and a low level of coherence. Therefore, both categories require many complex skills, and these skills are heterogeneous and unrelated to each other. However, this similar skill composition is not reflected in the distribution of wages, which rewards managerial occupations substantially more. In light of the observed relations between fitness and wages, the relationship between AC and average yearly wages in the right panel of Fig. 5 appears also decreasing but L-shaped with two more extreme emerging patterns. Jobs with low coherence (AC $\lesssim 0.45$) show highly heterogeneous wages, ranging from 30,000 to 200,000 USD dollars, with e.g. “Life, physical and social science” occupations in the low-end and managerial occupations in the high-end. By contrast, for jobs with intermediate and high skill coherence (AC $\gtrsim$0.45), e.g. “Different clerical”, and production, installation, and maintenance occupations, it is not possible to obtain wages higher than 60,000 USD dollars, regardless of their degree of coherence.

### Complex jobs are abstract and non-routinary; coherent jobs are manual and routinary

The literature on job dynamics and polarization identifies different classes of jobs by using the typology of tasks they usually perform. By following Autor^65^, we divide jobs into routinary and non-routinary on the basis of the presence (or lack thereof) of codified sets of a finite number of simple tasks or procedures, and in manual or abstract if they require manual dexterity and physical strength or intuition and creativity, respectively.

In this subsection, we explore whether and to what extent job fitness and average job coherence, the two economic complexity dimensions introduced above, are connected to these qualitative and binary occupational characterisations by highlighting abstract/manual and routinary/non-routinary jobs in the scatter plots presented in Fig. 5.

**Figure 6:**
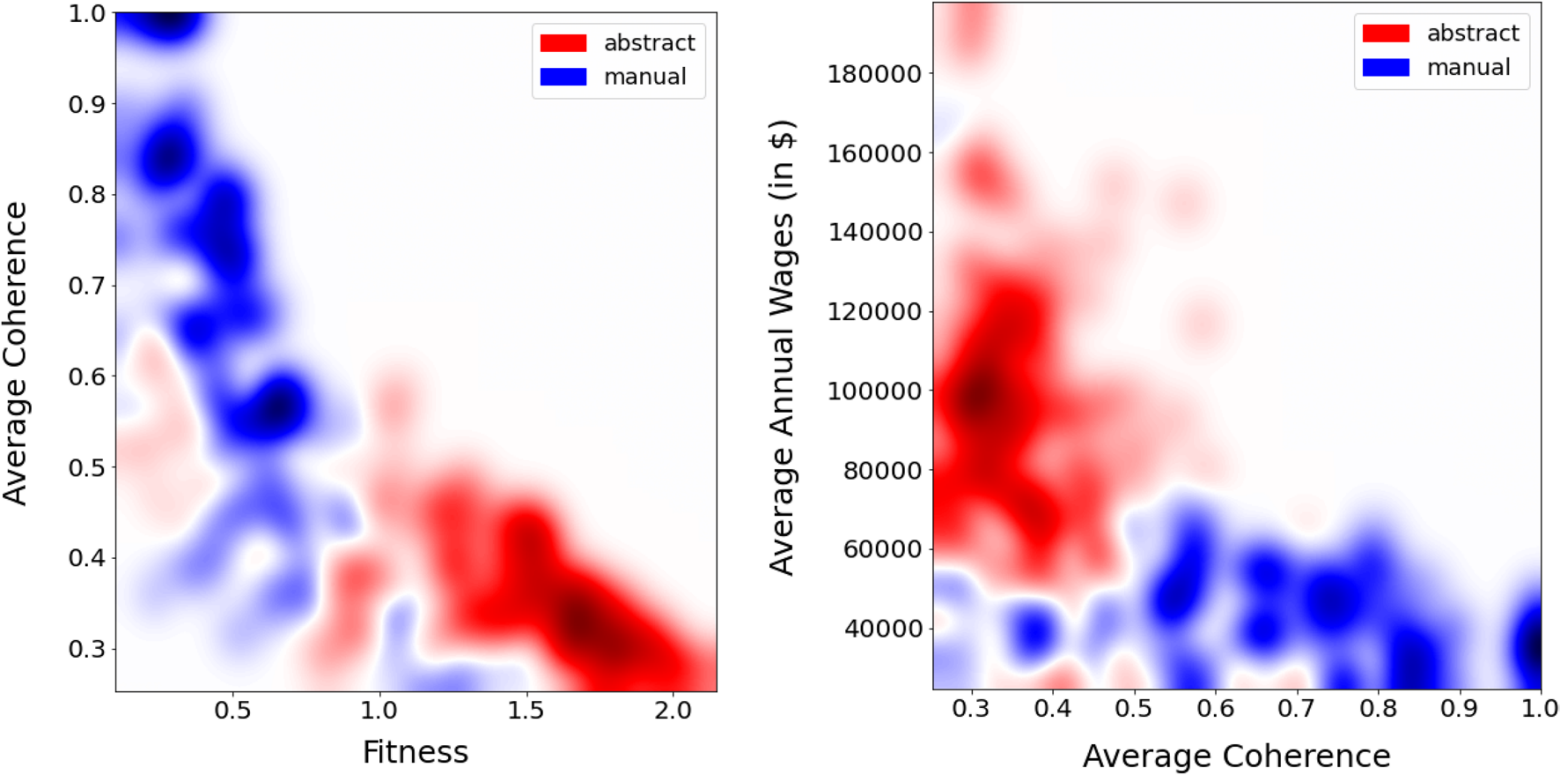
Heatmaps obtained from the scatter plots in Fig. 5. Jobs are represented as a function of their average coherence, fitness, and average wages. Colours correspond to their categorization in abstract (red) or manual (blue) occupations. High-fitness jobs are abstract, while highly coherent jobs are manual and present low wages.

**Figure 7:**
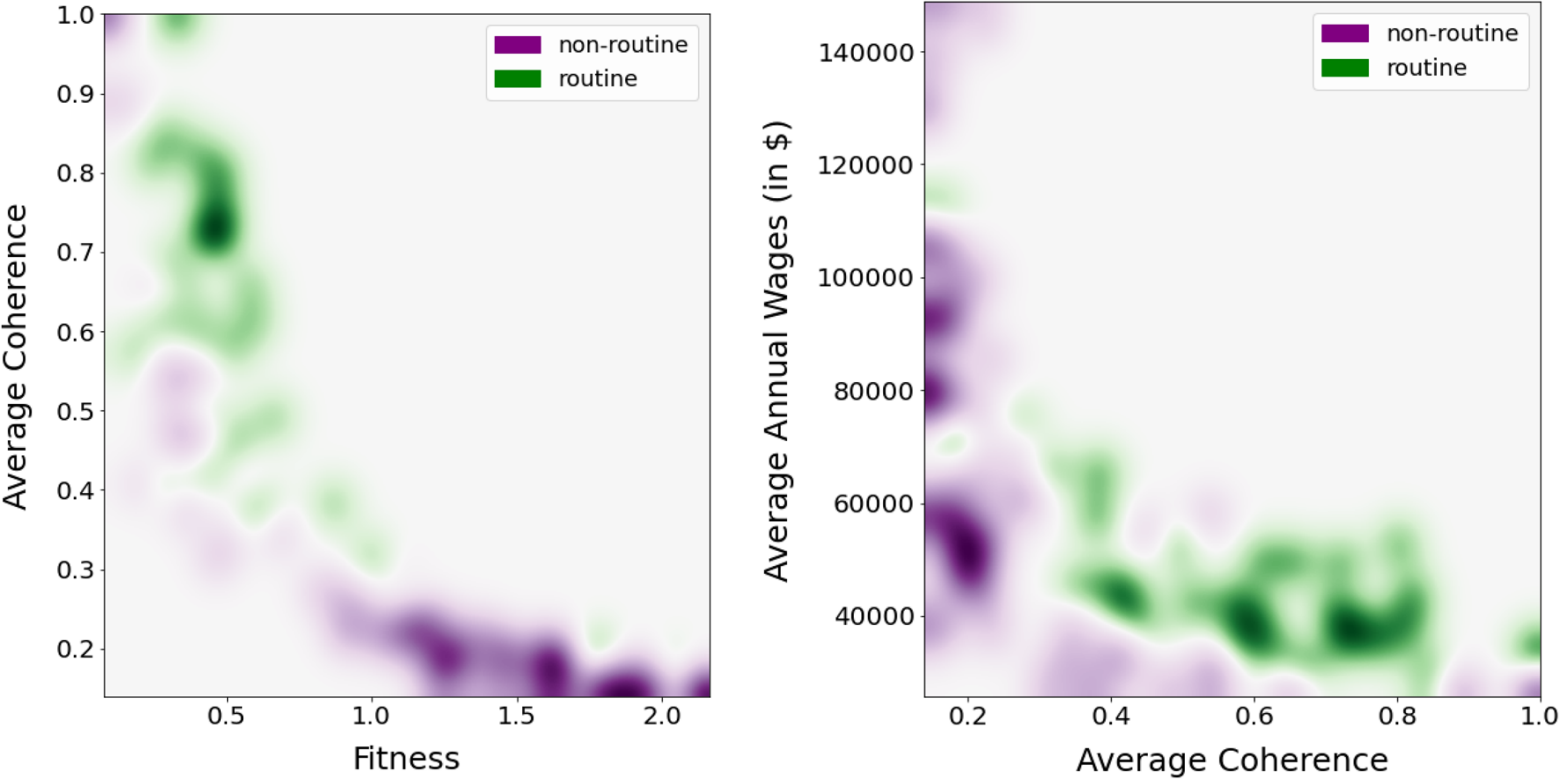
Heatmaps obtained from the scatter plots in Fig. 5. Jobs are represented as a function of their average coherence, fitness, and average wages. Colours correspond to their categorization in routinary (green) and non-routinary (purple) occupations. High-fitness jobs are non-routinary, while highly coherent jobs are mostly routinary and present low wages.

The heatmaps shown in Fig. 6 are obtained by smoothing the two scatter plots shown in Fig. 5, in which we coloured the dots in red if the corresponding jobs are abstract, and in blue if manual. The smoothing is performed using a Gaussian kernel (see the SciPy documentation of the *scipy.ndimage.gaussian_filter* library) with a kernel parameter *sigma* = 32. In such a way, we are able to visualize the density of job typologies in the plane defined by the Fitness-Average Coherence and Average Coherence-Average Annual Wages. In the AC-Fitness plane in the left panel it is possible to observe that the red abstract area is concentrated in the bottom right corner of the plot, where fitness is high and coherence is low, showing that high-fitness jobs are mainly abstract, while intermediate fitness jobs are a mixture of abstract and manual, and highly coherent jobs are prominently manual. Manual occupations in the top left corner of the figure include “Electrical and Electronics Assemblers”; “Machine Operators” or “Glaziers”. Examples of abstract jobs in the bottom right corner of the figure are “Secondary School Teachers”; “Fire Inspectors” or “Dentists”. In this area, we find a small number of manual jobs: “Pilots”; “Residential Advisors”; “Boat Operators”. Some abstract jobs, such as “Models”, “Billing and Posting Clerks”, and “Telemarketers” display low fitness and low average coherence. From the right panel, we can see that the L-shape of the AC-wage plot is further characterized when considering the distinction between abstract and manual jobs. Manual jobs occupy the horizontal band of the L-shaped plot: they are characterized by lower wages, below 70,000 USD dollars, and can have any level of coherence, also very low levels, shedding light on the low salaries of the low-coherence jobs in the vertical band. In contrast, high wages are available only to less coherent, abstract jobs in red in the upper vertical band.

In Fig. 7 we repeat this exercise, but here the colours correspond to routinary (green) and non-routinary (purple) occupations—where given the high class imbalance (the number of non-routinary jobs vastly exceeds the number of routinary jobs) of the Broad Occupations (431 jobs), we employ the Minor Groups (95 jobs). Looking at the left panel, we observe that the negative relationship between fitness and coherence is here split into two patterns: green in the left upper portion for routine occupations and for non-routine purple in the lower right corner. The high fitness-low coherence area is occupied mostly by non-routinary jobs—such as “Engineers”; “Postsecondary Teachers”, or “Top Executives”. Routinary jobs are instead characterized by high average coherence and low fitness—for instance, “Metal Workers”, “Office and Administrative Support Workers”, and “Financial Clerks”. There are however some deviations to this behaviour: the green points in the bottom right corner, which correspond to technical (supervision) jobs, i.e. “Supervisors of Production Workers”, “Supervisors of Installation”, “Maintenance and Repair Workers”, and “Air Transportation Workers”. While jobs with high coherence and low fitness are mainly routinary, some exceptions can be found, such as “Food and Beverage Serving Workers”; “Grounds Maintenance Workers”; “Entertainment Attendants”, that fall in the non-routinary class. In the right panel, we observe that high wages correspond to non-routinary and low-coherence jobs. With increasing values AC we note a clear lowering of the wages of non-routinary jobs, while routinary jobs with low coherence show relatively higher wages. Finally, low wages and low coherent jobs are mainly non-routinary.

While the classifications discussed above are binary (i.e., an occupation can only be manual or non manual) and somewhat arbitrary as they are obtained through expert evaluation, job fitness and average coherence are continuous metrics that (i) provide more nuanced and endogenous information, (ii) are algorithmic and data-driven and can thus help us formulate a meaningful index characterising each job based on its structural skill composition. The apparently clear linear relationship between job fitness, job AC, and the abstract/manual, routine/non-routine work taxonomies holds only on average, but sizeable deviations are found. Some of these deviations are already visible in Figs. 6 and 7, however in order to show this non-trivial relationship in a clearer way, we added to the Supplementary Information file the original scatter plots and a different version of the figures in which we used a lower smoothing parameter.

In conclusion, economic complexity measures can identify different typologies of jobs by extracting relevant information from the skill-job network. This information, such as the complexity content of skills and their coherence, is partially reflected in the abstraction and routinarity level of jobs, but, differently from these binary characterizations of jobs, it can be quantified, specifying, for instance, the degree of complexity for each skill and fitness and coherence for each job. Moreover, AC and fitness both show some interesting deviations from these binary classifications, thus helping us provide additional elements on the relationship between skill, tasks, and wages. Remarkably, non-routinarity is not enough to determine the wage level. Adding a network-based variable accounting for the skill coherence of each job allows us to observe that only high-fitness, low-coherent, abstract, non-routinary jobs are rewarded by high wages.

## Discussion

The progressive complexification of the world of work and increasing wage inequality in the globalised economy call for new tools beyond standard economics analysis to investigate the interplay between human capital and the occupational structure of the labour market. The combined availability of increasingly disaggregated data, algorithmic and complex network techniques allows us to unveil novel perspectives and results.

In this paper, by starting from the construction of a bipartite network connecting jobs to the skills they require, we apply different methods from the Economic Complexity toolbox to investigate the underlying network structure of occupations and skills in the US labour market relying on the information afforded by the O*NET data-set.

Firstly, we propose the definition of two statistically validated networks to qualify the structural similarities and relatedness between, respectively, pairs of skills and pairs of jobs: the job space and the skill space. In the job space, each node represents a job, and two jobs are connected if the skills they require co-occur in a significant (more often than random) number of jobs. In the skill space, each node corresponds to a skill, and two skills are linked if they co-occur significantly in multiple jobs. To build these networks we exploit the relatedness interpretation of the Product Progression Network methodology^27,56^, which, as mentioned, not only accounts for diversification and ubiquity in assessing network links but, differently from other approaches to relatedness^20^, proposes a links' statistical validation procedure, thus ensuring that only meaningful connections are considered and spurious links are filtered out^66^. The statistical significance of co-occurrences is a critical issue since the presence of a link may not be informative *per se* and could be simply due to the ubiquity of a skill or the diversification of a job. In particular, as in^35^, in this contribution, we assess the significance of links by using the Bipartite Configuration Model^59,67, 68^, a maximum entropy null-model designed to statistically validate bipartite network projections. The resulting network of skills shows two distinct communities, one including mainly scientific and technical skills and the other soft skills, humanities, and communication skills, with different business and management competencies acting as bridges between the two communities. A similarly segmented picture emerges from the job space, where, as confirmed by the community detection analysis reported in the SI, technical and manual occupations appear to be almost completely disconnected.

Second, we introduce a novel application of the Economic Fitness and Complexity algorithm to a bipartite network linking jobs to their skill and knowledge requirements and, for each job and skill in the O*NET dataset, we define a network-based measure of the inherent structural complexity of each occupation: job fitness—a measure of the degree of complexity in the skill mix of each occupation—and skill complexity—a measure of the sophistication and rarity of each skill or knowledge requirement. This application of the EFC algorithm to the job-skill network rests on the idea that a high fitness job requires a largely diversified skill set, comprising many complex skills but also more basic skills, while low complexity skills are required only by low-fitness jobs. The resulting rankings show that high-fitness jobs include, e.g., management and top executives, architects, and life scientists, but also some specific types of production or administrative occupations, while low-fitness jobs include, e.g., food preparation and serving workers, entertainment attendants, and administrative, maintenance or repair workers. Job fitness and skill complexity not only allow us to produce a fine-grained and continuous measure of the nature of occupations and skills, but are also endogenous metrics, as they do not rely on ex-post validations and can be informative about the relationship between the skill content of each job and average occupational wages. In fact, our approach adopts a data-driven perspective and refrains from presuming the existence of a direct technical production function relationship, and is thus a priori agnostic about the connection between wages and human capital. The identification of these complexity metrics may, in future works, also be the basis for producing an updated taxonomy of jobs and skills.

To further characterise what we have called the “human capabilities” necessary to obtain high job fitness, we leverage the concept of coherence proposed by Teece et al. and the statistically validated definition of relatedness by Zaccaria et al.^27,56^, and define a network-based measure of relatedness between skills that can be used to measure the average coherence (AC) of an occupation. AC is designed for assessing the degree of skill similarity within each occupation: coherent jobs require highly complementary skills, i.e. co-occurring more often than random in a large number of occupations. Average coherence provides a piece of information mainly negatively correlated to fitness: complex jobs present heterogeneous skill requirements, while low-fitness jobs present a highly coherent skill mix, characterized by a combination of related skills that are often demanded jointly.

Next, we offer a perspective on the endogenous determinants of occupational wages by examining the relationships between the complexity, coherence (or lack thereof) of a job’s skill set, and average occupational wages. We then compare our network-based measures with the specifications of manual/abstract work and routine/non-routine by means of a non-parametric analysis, showing that the degree of coherence and job fitness are related to these dichotomous and broad definitions but help provide a more granular characterization of skills. We detect an overall positive non-linear relationship between job fitness and occupational wages, with more complex skill requirements mapping into higher wages. However, our evidence shows noteworthy deviations from this positive trend, especially at the two extremes of the wage distribution, testifying to the presence of skill-independent wage-setting mechanisms. Firstly, jobs in the low-fitness area appear to be placed at the very bottom of the pay scale, in a low-wage trap apparently more driven by belonging to a specific occupation than the characteristics of the occupation’s skill requirements^41,51, 69^. Secondly and in line with the studies of top wage earners^50,64^, for the highest fitness jobs a decoupling between wages and the job’s skill complexity is observed. This is especially true for managerial occupations that, albeit showing similar skill profiles and fitness values with other business and financial occupations, exhibit higher wages and higher within-occupation wage differentials, also in this case apparently reflecting idiosyncratic characteristics more linked to their occupational status than their specific competences.

Job coherence also appears to be a potentially relevant factor for occupational wages, with higher coherence mapping into lower wages and a coherence threshold after which it is impossible to obtain a yearly wage higher than 60,000 USD. Low coherence jobs are instead spread across the occupational classification and display high wage variability, but, overall, workers with more diverse and complex skill bundles tend to earn higher wages than specialists, no matter the degree of routinarity. In fact, whilst high wages correspond to non-routinary, low-coherence, high-fitness jobs, low wages and low coherent jobs are mainly non-routinary: increasing a job’s skill coherence we register lower non-routinary wages, while routinary jobs with low coherence show relatively higher wages. High-fitness jobs tend to be abstract and low in coherence, while manual jobs are highly coherent and tend to have lower wages, with only low-wage low-coherence jobs being manual.

To sum up, differently from the variables’ definitions typically used in the network-based literature on job and skills spaces^10,40–42, 44–47^ discussed in the introduction, we employ a novel methodology for defining job fitness and skill complexity, and provide a sounder and statistically validated definition of occupational skill relatedness. This approach yields a richer quantitative description of the underlying complexity and coherence of occupational skill requirements, transcending conventional top–down job categorizations and offering insights into the wage implications of the structure of different skill sets. Additionally, by unpacking statistically significant links between pairs of jobs (skills) in the job (skill) network, our analysis may help trace potential sound career switching pathways based on the quantified degree of complementarity or mobility between single jobs (skills) with a high level of granularity. In conclusion, adopting the economic complexity lens enabled us to introduce complementary and alternative instruments to gain valuable structural insights into the occupational landscape and the heterogeneous distribution of skills in the labor market, also setting the basis for diverse labor market analyses. Indeed, given the existing data constraints, our analysis of the job and skill spaces is fixed in time. However, the skill content of jobs may change over time, across space^70,71^ or even between firms^72^. Therefore, provided the appropriate information—e.g., online job advertisement data as done by Deming and co-authors^70^—may open new possibilities for understanding the changing network structure of the occupational demand for skills, both in the time and space dimension. Job advertisement data, such as the Burning Glass technology database^73^ or obtained through web-scraping techniques, may also offer the possibility to analyse the demand side, similarly to^10^, or as potential catalysts for corporate performance by matching online job ads with micro business census data. Furthermore, it would be of interest to learn more about the role played by skill coherence and job fitness as drivers of occupational wages and in entry/exit dynamics in the labour market.

Finally, our findings may inform policymakers and employers on designing more effective labour market policies and on the job-training schemes, that should favor the acquisition of complex, cross-cutting, and “uncoherent” skill sets. These efforts may enable workers to move more easily throughout the job and skill spaces and make more informed choices about their professional paths while unlocking higher wage opportunities. At the same time, policy initiatives targeting the demand-side may aim at encouraging firms to make broader investments in a more diversified skill landscape rather than fostering hyper-specialization. These efforts, in fact, may also exert a favorable impact on firms’ growth and productivity, as wages can act as drivers of overall corporate performance^74–77^.

## Methods

### Data description and pre-processing

The Fitness and Complexity algorithm as well as the relatedness metrics introduced in the Results section take a binary bipartite network connecting jobs to their skill content as input. In this section, we illustrate the procedure to obtain the bipartite skill-job network from the raw O*NET database.

We retrieve information on occupations and skills from the Occupational Information Network (O*NET) (www.onetcenter.org), mantained by the US Department of Labor’s Employment and Training Administration (ETA). O*NET provides survey-based information about skills, knowledge, tasks, tools, and technologies connected to each job in the O*NET-SOC occupational classification^78^. The O*NET-SOC classification is hierarchical and contains different levels of aggregation. In particular, occupations, to which in the paper we refer also as jobs, are classified in the following categories:

- Detailed Occupations (873 categories);
- Broad Occupations (431 categories);
- Minor Groups (95 categories);
- Major Groups (22 categories).

We define our set of skills as the merge of skill and knowledge occupational requirements provided by O*NET, obtaining a total of 68 different skills. By following recent contributions in the Economic Complexity literature^22,79^, the combination of the occupational skills and knowledge attributes may provide a valuable basis for what we refer to as  “human capabilities”, providing an underlying foundation to the place-based notion of capabilities and mirroring the conceptual role productive capabilities play in the economic complexity literature. The choice to only include skill and knowledge variables and not education, the third component of the O*NET worker requirement section, is motivated by the fact that educational requirements provide a too broad classification for a complexity analysis, and we thus consider skill and knowledge requirements as manifestation of underlying education and training.

The skill and knowledge areas are, on average, diffused across all occupational categories, and the resulting bipartite matrix connecting those to occupations is characterized by a nested structure. For each Detailed Occupation and skill, O*NET provides an assessment of the *Importance* of the different skills. This is a discrete variable in the range [1, 5], quantifying the degree of importance of each skill for the job category under consideration—we neglect the other variable *Level* present in the dataset, as it is highly correlated with the importance. We thus obtain a matrix $\tilde{\textbf{M}}$, whose elements $\tilde{M}_{js}$ associate the importance of skill *s* to job *j* and where job categories are drawn from the 837 O*NET-SOC Detailed Occupations. Next, starting from matrix $\tilde{\textbf{M}}$, we build the matrices also for the more aggregated O*NET-SOC Broad occupations and Minor Groups by computing the importance of a given skill *s* for the aggregated category *k* as the weighted average importance of skill *s* in the corresponding Detailed Occupations that are aggregated into category *k*. In the following, we generally refer to these matrices as $\tilde{\textbf{M}}$ without referring to the occupation aggregation level.

Finally, the data about occupational wages is obtained from the Quarterly Census of Employment and Wages (QCEW) dataset of the US Bureau of labour Statistics (BLS, https://www.bls.gov/cew/), in which occupations are categorized according to the SOC classification.

#### Job-Skill binary matrix

Starting from matrix $\tilde{M}$ introduced above, we define a binary matrix *M* with the same size, but connecting jobs only to the most relevant corresponding skills, as all the EC tools we adopt in the analysis require binary matrices as inputs. In order to binarise the matrix we compute for each skill *s* its average importance in all occupations; then, for job *j* and skill *s*, we set the matrix entry  equal to 1 the entries corresponding to the jobs with importance greater than the average and equal to 0 otherwise. In formula:

$$\begin{aligned} M_{js}= {\left\{ \begin{array}{ll} 1\ \quad \text {if} \ \tilde{M}_{js}>\frac{1}{N^{(j)}}\sum _j\tilde{M}_{js}\\ 0\ \quad \text {otherwise} \end{array}\right. } \end{aligned}$$

where we denote by $N^{(j)}$ the total number of occupations.

### Null model and validation procedure

In order to build the monopartite networks of jobs and skills we compute the two respective projections of the bipartite matrix *M*. As explained in the Results section, this can be done using the adjacency matrices defined in Eq. (1), that is, in the case of the job network:

$$\begin{aligned} B^{Jobs}_{jj'}=\frac{1}{\max (d_j,d_{j'})}\sum _{s}\frac{M_{js}M_{j's}}{u_s} \end{aligned}$$

where $d_j=\sum _s M_{js}$ is the skill diversification of job *j*, i.e. the number of skills associated to *j*, and $u_s=\sum _j M_{js}$ is the ubiquity of skill *s*, i.e. the number of jobs in which *s* is present. In this way, by computing the normalized co-occurrences of skills across jobs, we obtain the similarity matrix $\textbf{B}^{Jobs}$ connecting jobs that share a large number of common skills. Analogously, we compute matrix $\textbf{B}^{Skills}$, whose elements $B^{Skills}_{ss'}$ quantify the similarity between skill *s* and skill $s'$ in terms of normalized co-occurrences. However, such an adjacency matrix generally corresponds to an almost fully connected network due to spurious co-occurrences, therefore it is necessary to statistically filter the matrix links by exploiting a suitable null model. To this aim, we rely on the Bipartite Configuration Model (BiCM)^59,67, 80^, a null model designed to randomize bipartite networks based on exponential random graph theory. In particular, we rely on the Python BiCM library (https://bipartite-configuration-model.readthedocs.io/en/latest/). BiCM defines a canonical ensemble of random graphs by constraining (on average) the degree sequences of both node sets—in our case, the ubiquity of skills and the diversification of jobs. We obtain the probability distribution of such an ensemble by maximizing the Shannon entropy under these constraints in the following way:

$$\begin{aligned} P \left( \bar{\textbf{M}}|\{\theta _j\}, \{\mu _s\} \right) = \frac{{{\,\textrm{e}\,}}^{-H \left( \bar{\textbf{M}}|\{\theta _j\}, \{\mu _s\} \right) }}{Z \left( \{\theta _j\}, \{\mu _s\} \right) }, \end{aligned}$$

where:

- $\bar{\textbf{M}}$ is the adjacency matrix of the random bipartite network;
- $\{\theta _j\}$ and $\{\mu _s\}$ are the Lagrange multipliers associated respectively to the diversification $\{d_j\}$ and the ubiquity $\{u_s\}$;
- $H (\bar{\textbf{M}}|\{\theta _j\}, \{\mu _s\})$ is the Hamiltonian, defined as:
  $$\begin{aligned} H(\bar{\textbf{M}}|\{\theta _j\}, \{\mu _s\})= \sum _j \theta _j d_j(\bar{\textbf{M}}) + \sum _s \mu _s u_s(\bar{\textbf{M}}); \end{aligned}$$
- $Z(\{\theta _j\}, \{\mu _s\})$ is the partition function of the Hamiltonian, defined as:
  $$\begin{aligned} Z \left( \{\theta _j\}, \{\mu _s\} \right) =\sum _{\bar{\textbf{M}}}{{\,\textrm{e}\,}}^{-H \left( \bar{\textbf{M}}|\{\theta _j\}, \{\mu _s\} \right) }. \end{aligned}$$

It can be shown that the probability distribution factorizes and takes the analytical form:

$$\begin{aligned} P \left( \bar{\textbf{M}}|\{\theta _j\}, \{\mu _s\} \right) =\prod _{j, s}p_{js}^{\bar{M}_js}(1-p_{js})^{1-\bar{M}_{js}} \end{aligned}$$

where $p_{js}$ is the probability that job *j* and skill *s* are connected in the random network and satisfies the following condition:

$$\begin{aligned} p_{js} = \frac{1}{{{\,\textrm{e}\,}}^{\theta _i+\mu _j}+1}. \end{aligned}$$

The numerical values of the Lagrange multipliers are obtained by solving the system of equations:

$$\begin{aligned} {\left\{ \begin{array}{ll} d_j = \sum_s p_{js}\\ u_s = \sum_j p_{js}. \end{array}\right. } \end{aligned}$$

Once we obtain the probability distribution of the random matrices and, in particular, the probability of each link's presence in the adjacency matrix of the job-skill network, we can generate *N* random bipartite networks, and, for each of them, we compute the projected matrix $\bar{\textbf{B}}^{Jobs}$ defined above. We then validate the links of $\textbf{B}^{Jobs}$ by only considering the matrix entries that are larger than the corresponding entries in the random matrices in at least the $95\%$ of the cases, while we set to zero the remaining links. Where the $95\%$ statistical significance threshold is arbitrary and sets the chosen confidence level. To obtain the validated monopartite network of skills, we apply the same procedure to matrix $\textbf{B}^{Skills}$.

### Economic fitness and complexity algorithm

The Economic Fitness and Complexity (EFC) algorithm^11^ is an iterative and non-linear map initially introduced to study the Country-Product bipartite network based on international trade data. The algorithm outputs the Fitness of countries, an proxy for a country’s manufacturing capabilities and productive competitiveness, and the Complexity of products which quantifies the sophistication and rareness of goods. The EFC algorithm has been successfully applied in types of different bipartite networks, focusing on the scientific^81^, technological^82^ or sectoral production of regions or countries^18^, to rank species in mutualistic networks^83^ or chess players^84^. In the present paper, we apply the EFC algorithm to the Job-Skill bipartite network and compute for each job *j* a measure of Fitness $F_j$ by counting how many skills it requires and weighting each skill *s* by its complexity $Q_s$. Therefore, in this context, high-fitness jobs are skill-diversified and/or require complex skills. For each skill *s* we compute a complexity measure $Q_s$ by considering the inverse of the number of jobs requiring *s* and weighting each job with the inverse of its fitness $F_j$, in such a way, a skill present in a large number of jobs or required only in low fitness jobs will display low complexity. Therefore, the non-linear map which defines the $F_j$s and the $Q_s$ can be written as follows:

$$\begin{aligned} {\left\{ \begin{array}{ll} \tilde{F}_j^{(n)} = \sum _s M_{js}Q_s^{(n-1)}\\ \tilde{Q}_s^{(n)} = \frac{1}{\sum _j M_{js}\frac{1}{F_j^{(n)}}} \end{array}\right. }\quad \quad {\left\{ \begin{array}{ll} F_j^{(n)} = \frac{\tilde{F}_j^{(n)}}{\langle \tilde{F}_j^{(n)}\rangle _j}\\ Q_s^{(n)} = \frac{\tilde{Q}_s^{(n)}}{\langle \tilde{Q}_s^{(n)}\rangle _s} \end{array}\right. } \end{aligned}$$

where we denote with *n* the algorithm iteration index and consider $Q_s=1$ for all skills as initial condition. The iteration of these equations leads to a fixed point that has been proven to be stable and non-dependent on initial conditions^60^. We stop the iteration of the algorithm when the ranking of job fitness becomes stable, exploiting the technique described in^61^. To this aim, we firstly define the fitness growth rate $\alpha _j$ of job *j* as:

$$\begin{aligned} \alpha _j = \frac{\log \left( F_j^{(n)}-\log \left( F_j^{(n-2)} \right) \right) }{\log (n)-\log (n-2)}. \end{aligned}$$

Secondly, we estimate when job *j* and job $j+1$ will exchange their position in the fitness ranking as:

$$\begin{aligned} CI_j = n\left( \frac{F_j^{(n)}}{F_{j+1}^{(n)}}\right) ^{\frac{1}{\alpha_{j+1}-\alpha _j}}. \end{aligned}$$

Notice that we preliminary ordered the jobs so that $F_j^{(n)}>F_{j+1}^{(n)}$ and $CI_j$ is well defined only if $\alpha _{j+1}-\alpha _j>0$. Among these well-defined $CI_j$, we define the Minimum Crossing Iteration as:

$$\begin{aligned} MCI^{(n)}=\min _c(CI_j) \end{aligned}$$

which indicates the number of iterations needed to observe a variation. Thus, imposing a convergence in the ranking here is equivalent to requesting an MCI larger than a threshold which we fix equal to $10^6$.

Please notice that peculiar topologies of the input matrix ${\textbf {M}}$ may give rise to unexpected outputs of the algorithm. In particular, disconnected components prevent the possibility of comparing the rankings of the nodes that belong to different clusters, and the presence of monopolies induces a high advantage in the final rankings. Two examples are “First-Line Supervisors of Firefighting and Prevention Workers” and “Morticians, Undertakers, and Funeral Arrangers”: the algorithm assigns them a high level of Fitness due to their specialized and uncommon skills. For a critical discussion on these issues we refer to^61^.

### Supplementary Information

Supplementary Information.

Supplementary Information.

Supplementary Information.

Supplementary Information.

Supplementary Information.

## References

[1] Acemoglu, D. & Restrepo, P. *Artificial Intelligence* (Automation and Work. Tech. Rep, National Bureau of Economic Research, 2018).

[2] Frey, C. B. & Osborne, M. A. The future of employment: How susceptible are jobs to computerisation?. *Technol. Forecast. Soc. Change* **114**, 254–280 (2017). 10.1016/j.techfore.2016.08.019

[3] Barbieri, N. & Consoli, D. Regional diversification and green employment in us metropolitan areas. *Res. Policy* **48**, 693–705 (2019). 10.1016/j.respol.2018.11.001

[4] Lankhuizen, M., Diodato, D., Weterings, A., Ivanova, O. & Thissen, M. Identifying labour market bottlenecks in the energy transition: A combined io-matching analysis. *Econ. Syst. Res.* **35**, 157–182 (2023). 10.1080/09535314.2022.2048294

[5] Rughi, T., Staccioli, J. & Virgillito, M. E. Climate change and labour-saving technologies: The twin transition via patent texts. *Available SSRN 4407851* (2023).

[6] Brynjolfsson, E. & Milgrom, P. *Complementarity in Organizations* (Princeton University Press, 2013).

[7] Deming, D. J. The growing importance of social skills in the labor market. *Q. J. Econ.* **132**, 1593–1640 (2017). 10.1093/qje/qjx022

[8] Gathmann, C. & Schönberg, U. How general is human capital? A task-based approach. *J. Law Econ.* **28**, 1–49 (2010).

[9] Woolley, A. W., Chabris, C. F., Pentland, A., Hashmi, N. & Malone, T. W. Evidence for a collective intelligence factor in the performance of human groups. *Science* **330**, 686–688 (2010). 20929725 10.1126/science.1193147

[10] Anderson, K. A. Skill networks and measures of complex human capital. *Proc. Natl. Acad. Sci.* **114**, 12720–12724 (2017). 29133397 10.1073/pnas.1706597114 PMC5715742

[11] Tacchella, A., Cristelli, M., Caldarelli, G., Gabrielli, A. & Pietronero, L. A new metrics for countries’ fitness and products’ complexity. *Sci. Rep.* **2**, 723 (2012). 23056915 10.1038/srep00723 PMC3467565

[12] Hidalgo, C. A. & Hausmann, R. The building blocks of economic complexity. *Proceed. nat. acad. sci.* **106**(26), 10570–10575 (2009). 10.1073/pnas.0900943106 PMC2705545 19549871

[13] Frenken, K. & Boschma, R. A. A theoretical framework for evolutionary economic geography: Industrial dynamics and urban growth as a branching process. *J. Econ. Geogr.* **7**, 635–649 (2007). 10.1093/jeg/lbm018

[14] Adam, A., Garas, A., Katsaiti, M.-S. & Lapatinas, A. Economic complexity and jobs: An empirical analysis. *Econ. Innov. New Technol.* **32**, 1–28 (2021).

[15] Basile, R., Cicerone, G. & Iapadre, L. Economic complexity and regional labor productivity distribution: Evidence from Italy. *Rev. Reg. Stud.* 201–219 (2019).

[16] Caldarola, B. Structural change (s) in Ghana: A comparison between the trade, formal and informal sectors. *Lab. Econ. Manag. (LEM) Work. Pap. Ser.* (2022).

[17] Fritz, B. S. & Manduca, R. A. The economic complexity of US metropolitan areas. *Reg. Stud.* **55**, 1299–1310 (2021). 10.1080/00343404.2021.1884215

[18] Sbardella, A., Pugliese, E. & Pietronero, L. Economic development and wage inequality: A complex system analysis. *PLoS ONE* **12**, e0182774 (2017). 28926577 10.1371/journal.pone.0182774 PMC5604954

[19] Boschma, R. & Frenken, K. *Evolutionary Economic Geography* (Oxford University Press, 2018).

[20] Hidalgo, C. A., Klinger, B., Barabási, A.-L. & Hausmann, R. The product space conditions the development of nations. *Science* **317**, 482–487 (2007). 17656717 10.1126/science.1144581

[21] Costa, S. *et al.* From organizational capabilities to corporate performances: At the roots of productivity slowdown. *Ind. Corp. Change* (2023).

[22] Diodato, D., Hausmann, R. & Schetter, U. A simple theory of economic development at the extensive industry margin. HKS Working Paper No. RWP22-016 (2022).

[23] Dosi, G., Faillo, M. & Marengo, L. Organizational capabilities, patterns of knowledge accumulation and governance structures in business firms: An introduction. *Organ. Stud.* **29**(8–9), 1165–1185 (2008). 10.1177/0170840608094775

[24] Neffke, F. & Henning, M. Skill relatedness and firm diversification. *Strateg. Manag. J.* **34**, 297–316 (2013). 10.1002/smj.2014

[25] Nelson, R. R. & Winter, S. G. *An Evolutionary Theory FO Economic Change* (Harvard University Press, 1982).

[26] Penrose, E. *The Theory of the Growth of the Firm* (Oxford University Press, 1995).

[27] Zaccaria, A., Cristelli, M., Tacchella, A. & Pietronero, L. How the taxonomy of products drives the economic development of countries. *PLoS ONE* **9**, e113770 (2014). 25486526 10.1371/journal.pone.0113770 PMC4259464

[28] Neffke, F., Henning, M. & Boschma, R. How do regions diversify over time? Industry relatedness and the development of new growth paths in regions. *Econ. Geogr.* **87**, 237–265 (2011). 10.1111/j.1944-8287.2011.01121.x

[29] Boschma, R., Minondo, A. & Navarro, M. The emergence of new industries at the regional level in Spain: A proximity approach based on product relatedness. *Econ. Geogr.* **89**, 29–51 (2013). 10.1111/j.1944-8287.2012.01170.x

[30] Breschi, S., Lissoni, F. & Malerba, F. Knowledge-relatedness in firm technological diversification. *Res. Policy* **32**, 69–87 (2003). 10.1016/S0048-7333(02)00004-5

[31] Boschma, R., Balland, P.-A. & Kogler, D. F. Relatedness and technological change in cities: The rise and fall of technological knowledge in US metropolitan areas from 1981 to 2010. *Ind. Corp. Change* **24**, 223–250 (2014). 10.1093/icc/dtu012

[32] Napolitano, L., Evangelou, E., Pugliese, E., Zeppini, P. & Room, G. Technology networks: The autocatalytic origins of innovation. *R. Soc. Open Sci.* **5**, 172445 (2018). 30110482 10.1098/rsos.172445 PMC6030307

[33] Barbieri, N. *et al.* Regional technological capabilities and green opportunities in Europe. *J. Technol. Transf.* **48**, 1–30 (2022).

[34] de Cunzo, F., Petri, A., Zaccaria, A. & Sbardella, A. The trickle down from environmental innovation to productive complexity. *Sci. Rep.* **12**(1), 22141 (2022). 10.1038/s41598-022-25940-6 PMC9780348 36550185

[35] Pugliese, E. *et al.* Unfolding the innovation system for the development of countries: Coevolution of science, technology and production. *Sci. Rep.* **9**, 1–12 (2019). 31712700 10.1038/s41598-019-52767-5 PMC6848202

[36] Galetti, J. R. B., Tessarin, M. S. & Morceiro, P. C. Skill relatedness, structural change and heterogeneous regions: Evidence from a developing country. *Pap. Reg. Sci.* **100**, 1355–1376 (2021). 10.1111/pirs.12629

[37] Maliranta, M. & Nikulainen, T. *Labour Force Paths as Industry Lingages: A Perspective on Clusters and Industry Life Cycles. Tech. Rep, ETLA Discussion Papers* (2008).

[38] Landman, M., Ojanperä, S., Kinsella, S. & O’Clery, N. The role of relatedness and strategic linkages between domestic and MNE sectors in regional branching and resilience. *J. Technol. Transf.* **48**, 1–45 (2022).

[39] O’Clery, N. & Kinsella, S. Modular structure in labour networks reveals skill basins. *Res. Policy* **51**, 104486 (2022). 10.1016/j.respol.2022.104486

[40] del Rio-Chanona, R. M., Mealy, P., Beguerisse-Díaz, M., Lafond, F. & Farmer, J. D. Occupational mobility and automation: A data-driven network model. *J. R. Soc. Interface* **18**, 20200898 (2021). 33468022 10.1098/rsif.2020.0898 PMC7879770

[41] Villarreal, A. The US occupational structure: A social network approach. *Sociol. Sci.* **7**, 187–221 (2020). 10.15195/v7.a8

[42] Axtell, R. L., Guerrero, O. A. & López, E. Frictional unemployment on labor flow networks. *J. Econ. Behav. Organ.* **160**, 184–201 (2019). 10.1016/j.jebo.2019.02.028

[43] López, E., Guerrero, O. A. & Axtell, R. L. A network theory of inter-firm labor flows. *EPJ Data Sci.* **9**, 1–41 (2020). 10.1140/epjds/s13688-020-00251-w

[44] Hartmann, D., Jara-Figueroa, C., Kaltenberg, M. & Gala, P. *Mapping Stratification: The Industry-occupation Space Reveals the Network Structure of Inequality. Hohenheim Discussion Papers in Business, Economics and Social Sciences* (2019).

[45] Muneepeerakul, R., Lobo, J., Shutters, S. T., Goméz-Liévano, A. & Qubbaj, M. R. Urban economies and occupation space: Can they get “there’’ from “here’’?. *PLoS ONE* **8**, e73676 (2013). 24040021 10.1371/journal.pone.0073676 PMC3767603

[46] Farinha, T., Balland, P.-A., Morrison, A. & Boschma, R. What drives the geography of jobs in the US? Unpacking relatedness. *Ind. Innov.* **26**, 988–1022 (2019). 10.1080/13662716.2019.1591940

[47] Alabdulkareem, A. *et al.* Unpacking the polarization of workplace skills. *Sci. Adv.* **4**, eaao6030 (2018). 30035214 10.1126/sciadv.aao6030 PMC6051733

[48] Pietronero, L. *et al.* Economic complexity: “Buttarla in caciara” vs a constructive approach. *arXiv preprint* arXiv:1709.05272 (2017).

[49] Mariani, M. S., Vidmer, A., Medo, M. & Zhang, Y.-C. Measuring economic complexity of countries and products: Which metric to use?. *Eur. Phys. J. B* **88**, 1–9 (2015). 10.1140/epjb/e2015-60298-7

[50] Atkinson, A. B., Piketty, T. & Saez, E. Top incomes in the long run of history. *J. Econ. Lit.* **49**, 3–71 (2011). 10.1257/jel.49.1.3

[51] Weeden, K. A. & Grusky, D. B. The case for a new class map. *Am. J. Sociol.* **111**, 141–212 (2005). 10.1086/428815

[52] Teece, D. J., Rumelt, R., Dosi, G. & Winter, S. Understanding corporate coherence: Theory and evidence. *J. Econ. Behav. Organ.* **23**, 1–30 (1994). 10.1016/0167-2681(94)90094-9

[53] Hidalgo, C. A. *et al.* The principle of relatedness. In *International Conference on Complex Systems* 451–457 (Springer, 2018).

[54] Tacchella, A., Zaccaria, A., Miccheli, M. & Pietronero, L. Relatedness in the era of machine learning. *Chaos Solitons Fractals* **176**, 114071 (2023). 10.1016/j.chaos.2023.114071

[55] Pugliese, E., Napolitano, L., Zaccaria, A. & Pietronero, L. Coherent diversification in corporate technological portfolios. *PloS One* **14**, e0223403 (2019). 31600259 10.1371/journal.pone.0223403 PMC6786614

[56] Zaccaria, A., Mishra, S., Cader, M. Z. & Pietronero, L. Integrating services in the economic fitness approach. *World Bank Policy Res. Work. Paper* (2018).

[57] Mariani, M. S., Ren, Z.-M., Bascompte, J. & Tessone, C. J. Nestedness in complex networks: Observation, emergence, and implications. *Phys. Rep.* **813**, 1–90 (2019). 10.1016/j.physrep.2019.04.001

[58] Albora, G. & Zaccaria, A. Machine learning to assess relatedness: The advantage of using firm-level data. *Complexity* **2022**, 2095048 (2022). 10.1155/2022/2095048

[59] Saracco, F. *et al.* Inferring monopartite projections of bipartite networks: An entropy-based approach. *New J. Phys.* **19**, 053022 (2017). 10.1088/1367-2630/aa6b38

[60] Cristelli, M., Gabrielli, A., Tacchella, A., Caldarelli, G. & Pietronero, L. Measuring the intangibles: A metrics for the economic complexity of countries and products. *PLoS ONE* **8**, e70726 (2013). 23940633 10.1371/journal.pone.0070726 PMC3733723

[61] Pugliese, E., Zaccaria, A. & Pietronero, L. On the convergence of the fitness-complexity algorithm. *Eur. Phys. J. Special Top.* **225**, 1893–1911 (2016). 10.1140/epjst/e2015-50118-1

[62] Zaccaria, A., Cristelli, M., Kupers, R., Tacchella, A. & Pietronero, L. A case study for a new metrics for economic complexity: The Netherlands. *J. Econ. Interact. Coord.* **11**, 151–169 (2016). 10.1007/s11403-015-0145-9

[63] Bransden, B. H. & Joachain, C. J. *Physics of Atoms and Molecules* (Pearson Education India, 2003).

[64] Mishel, L. & Davis, A. Top CEOs make 300 times more than typical workers. *Econ. Policy Inst.* **21**, 1–14 (2015).

[65] Autor, D. H. & Dorn, D. The growth of low-skill service jobs and the polarization of the US labor market. *Am. Econ. Rev.* **103**, 1553–97 (2013). 10.1257/aer.103.5.1553

[66] Cimini, G., Carra, A., Didomenicantonio, L. & Zaccaria, A. Meta-validation of bipartite network projections. *Commun. Phys.* **5**, 1–12 (2022). 10.1038/s42005-022-00856-9

[67] Saracco, F., Di Clemente, R., Gabrielli, A. & Squartini, T. Randomizing bipartite networks: The case of the World Trade Web. *Sci. Rep.* **5**, 10595 (2015). 26029820 10.1038/srep10595 PMC4450581

[68] Vallarano, N. *et al.* Fast and scalable likelihood maximization for exponential random graph models with local constraints. *Sci. Rep.* **11**, 1–33 (2021). 34315920 10.1038/s41598-021-93830-4 PMC8316481

[69] Cetrulo, A., Sbardella, A. & Virgillito, M. E. Vanishing social classes? Facts and figures of the Italian labour market. *J. Evol. Econ.* **33**, 1–52 (2022). 36415649 10.1007/s00191-022-00793-4 PMC9668399

[70] Deming, D. & Kahn, L. B. Skill requirements across firms and labor markets: Evidence from job postings for professionals. *J. Law Econ.* **36**, S337–S369 (2018).

[71] Feng, X. & Rutherford, A. The dynamic resilience of urban labour networks. *arXiv preprint* arXiv:2202.12856 (2022). 10.1098/rsos.230214 PMC10320346 37416825

[72] De Marzo, G., Mathew, N. & Sbardella, A. *Who Creates Jobs with Broad Skillsets? The Crucial Role of Firms. Tech. Rep, International Labour Organization* (2023).

[73] Lancaster, V., Mahoney-Nair, D. & Ratcliff, N. J. *Technology Report Review of Burning Glass Job-Ad Data* (University of Virginia, Biocomplexity Institute and Initiative Social and Decision Analytics Division, 2019).

[74] Cesaratto, S., Serrano, F. & Stirati, A. Technical change, effective demand and employment. *Rev. Polit. Econ.* **15**, 33–52 (2003). 10.1080/09538250308444

[75] Cetrulo, A., Guarascio, D. & Virgillito, M. E. Anatomy of the Italian occupational structure: Concentrated power and distributed knowledge. *Ind. Corp. Change* **29**, 1345–1379 (2020). 10.1093/icc/dtaa050

[76] Fanti, L., Guarascio, D. & Tubiana, M. Skill mismatch and the dynamics of Italian companies’ productivity. *Appl. Econ.* **53**, 6790–6803 (2021). 10.1080/00036846.2021.1948963

[77] Winter, S. G. Knowledge and competence as strategic assets. In *The Strategic Management of Intellectual Capital* 165–187 (Routledge, London, 2009).

[78] Gregory, C., Lewis, P., Frugoli, P. & Nallin, A. Updating the O* NET-SOC taxonomy: Incorporating the 2018 SOC structure. *National Center O* NET Dev.* https://www.onetcenter.org/reports/Taxonomy2019.html (2019).

[79] Diodato, D., Morrison, A. & Petralia, S. Migration and invention in the age of mass migration. *J. Econ. Geogr.* **22**, 477–498 (2022). 10.1093/jeg/lbab032

[80] Squartini, T. & Garlaschelli, D. Analytical maximum-likelihood method to detect patterns in real networks. *New J. Phys.* **13**, 083001 (2011). 10.1088/1367-2630/13/8/083001

[81] Cimini, G., Gabrielli, A. & Labini, F. S. The scientific competitiveness of nations. *PLoS ONE* **9**, e113470 (2014). 25493626 10.1371/journal.pone.0113470 PMC4262272

[82] Sbardella, A., Perruchas, F., Napolitano, L., Barbieri, N. & Consoli, D. Green technology fitness. *Entropy* **20**, 776 (2018). 33265864 10.3390/e20100776 PMC7512338

[83] Domínguez-García, V. & Munoz, M. A. Ranking species in mutualistic networks. *Sci. Rep.* **5**, 1–7 (2015). 10.1038/srep08182 PMC4313099 25640575

[84] De Marzo, G. & Servedio, V. D. Quantifying the complexity and similarity of chess openings using online chess community data. *arXiv preprint* arXiv:2206.14312 (2022). 10.1038/s41598-023-31658-w PMC10067813 37005474

