# Supplementary material for: Mapping job fitness and skill coherence into wages: an economic complexity analysis
Source: Sci Rep. 2024 May 23;14:11752. doi: 10.1038/s41598-024-61448-x (PMC11116373; doi:10.1038/s41598-024-61448-x)
Supplement: Supplementary file 2 — Supplementary Information. [file 41598_2024_61448_MOESM2_ESM.pdf]

Mapping job complexity and skills into wages: Supplementary Information

Sabrina Aufiero[1, 2], Giordano De Marzo[1,3,4,5], Angelica Sbardella[3*], and Andrea Zaccaria[6,3]

[1]Dipartimento di Fisica Università "Sapienza", P.le A. Moro, 2, I-00185 Rome, Italy
[2]Department of Computer Science University College London, 66-72 Gower St, London WC1E 6EA, UK
[3]Centro Ricerche Enrico Fermi, Piazza del Viminale, 1, I-00184 Rome, Italy.
[4]Complexity Science Hub Vienna, Josefstaedter Strasse 39, 1080, Vienna, Austria.
[5]Sapienza School for Advanced Studies, "Sapienza", P.le A. Moro, 2, I-00185 Rome, Italy
[6]Istituto dei Sistemi Complessi (ISC) - CNR, UoS Sapienza,P.le A. Moro, 2, I-00185 Rome, Italy.
[*]angelica.sbardella@cref.it

June 18, 2024

1 Networks description and analysis

1.1 Network of Jobs

Here we provide a more detailed description of the Job Network. In the bottom right corner we find a highly connected community composed by almost all manual/technical jobs present in the dataset, from construction-related to fishing and hunting occupations. These kinds of jobs share many skills (for instance *"Production and Processing"*, *"Building and Construction"*, *"Mechanical"*, *"Equipment Selection"*, *"Operation Monitoring"*), therefore it might be relatively easy to move from the first to the second group leveraging the similarly required competences. By contrast, they are also isolated from other high-skill services and more abstract jobs, highlighting a clear dichotomy in the job landscape and, except for a few external nodes, low chances of moving out of the community. On the left of the upper community, in pink, we find healthcare occupations. These show a number of common skills related to the medical world or to the interaction with people such as *"Therapy and Counseling"*, *"Customer and Personal Service"*, and *"Psychology"*. On the top of the upper community are placed administration and sales occupations – in yellow and characterized by *"Administration and Management"*, *"Education and Training"*, and *"Economics and Accounting"* as common skills –, and managerial and professional occupations (green and orange respectively). These two groups are very similar in terms of the skills they share, the latter differing in the presence of problem-solving related skills such as *"Critical Thinking"* and *"Learning Strategies"*. "Food preparation and serving" – "Personal Services" in lime green – are more isolated, indicating poor possibilities of retraining due to a high level of specialization in a set of skills hardly useful elsewhere.

Finally, to better understand the role played by each occupation in the network and the possible connections between the communities, we quantify the *betweenness* of jobs. This is a measure of centrality that computes the number of shortest paths between all pairs of jobs in the network, displaying high scores for job nodes that act as bridges connecting different occupational communities. In the network of jobs we have constructed, we observe two distinct groups of jobs with high betweenness. The first group (in the right portion of the plot) is composed of scientific occupations, e.g., "Computer Occupations", "Physical Scientists", "Engineers", featuring *"Physics"*, *"Programming"*, *"Mathematics"*, and *"Complex problem solving"* as common skills. In the left portion of the plot, we find the second group, which includes mainly manual

and technical occupations – e.g., "Motor vehicle operators", "Cooks and Food Preparation" and "Protective Technicians" featuring *"Public Safety and Security"*, *"Law and Government"*, *"Telecommunications"*, and *"Monitoring"* as common skills.

1.2 Network of Skills

Here we provide a more detailed description of the Skill Network. The skills composing the first scientific-technical community range from fundamental scientific skills such as *"Physics"* – which occupies a central position featuring links with almost all the other nodes in the community –, *"Chemistry"*, high-skill technical competences such as *"Technology Design"*, *"Engineering and Technology"*, to lower-skill technical competences such as *"Installation"*, *"Construction"*, *"Mechanics"*, and *"Equipment Selection"*, *"Equipment maintenance"*, *"Operation and control"* etcetera. In contrast, the abstract skill community exhibits a combination of basic competencies – e.g., *"Speaking"*, *"Writing"*, *"Critical thinking"* and *"Mathematical knowledge"* – mainly positioned at the core of the community, with different cross-functional skills such as *"Social perceptiveness"*, *"Decision making"*, *"Problem solving"* and *"Coordination"*. Abstract and cross-functional skills appear to be essential inputs for different skills needed in a wide range of professional occupations covering all arts and humanities, some health services such as *"Medicine and dentistry"* and *"Therapy and counseling"*, sciences such as *"Psychology"*, *"Biology"*, and *"Geography"*, education, law, and communication-related knowledge. As mentioned above, *"Computer and electronics"* is the only technical skill disjointed from the first community, and it is densely connected with all basic skills and a large number of the cross-cutting skills present in the second community.

1.3 Community detection analysis

As we discussed in the main text and above, the two networks we obtained, the skill and job networks, both show the presence of communities. In order to better quantify this aspect, we applied different community detection algorithms to the networks, so to understand whether the communities emerging from a visual inspections are also recovered by this more rigorous approach. We exploit three different algorithms available in the NetworkX python library: greedy modularity maximization algorithm, Louvain algorithm and the asynchronous label propagation algorithm. It is well know that different algorithms can often return very different communities, testing several of them allows us to understand the reliability and strength of the communities we identified. Concerning the job-network, the result of the three different algorithms are very similar and stable over different random initial conditions. Both the greedy modularity maximization and the asynchronous label propagation almost always return two communities that coincide with those qualitatively identified in the main text. Conversely, Louvain algorithm tends to split the abstract jobs cluster into two separate communities. Moving to the skill-network, clusters appear to be less robust. The greedy modularity maximization algorithm always identifies three communities, one of them coinciding with the scientific and technical skills cluster we commented in the main text. Both Louvain and the asynchronous label propagation algorithms tend to find 4 or 5 communities, but again the technical skills community is always present. As an example of the results we obtained, we show if Fig. 1 the community structure obtained using the greedy modularity maximization algorithm. The conclusion of this analysis is that both networks present a very strong community, the technical skills community in one case and the manual jobs community in the other, while the remaining nodes are grouped in one or more communities depending on the specific algorithm.

2 Fitness tables

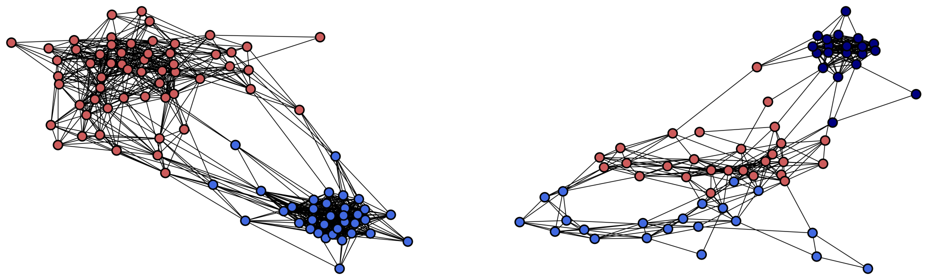

(a) (b)

Figure 1: a) Communities of the job network identified using the greedy modularity maximization algorithm. Different colors show the different communities identified. b) Communities of the skill network identified using the greedy modularity maximization algorithm.

Table 1: Ten Highest Fitness Jobs. These jobs stand out for their demand for specialized skills and their critical roles in various sectors.

**Job**	**Fitness**	**Average Annual Wages ($)**	**AC**
First-Line Supervisors of Firefighting and Prevention Workers	2.15	83.170	0.28
Conservation Scientists and Forester	2.05	68.120	0.28
Occupational Health and Safety Specialists and Technicians	2.05	74.480	0.28
Industrial Production Managers	2.04	118.190	0.28
Architects, Except Naval	2.02	87.130	0.28
Secondary School Teachers	1.98	67.240	0.3
Farmers, Ranchers and Other Agricultural Managers	1.97	76.810	0.28
Engineering and Architecture Teachers, Postsecondary	1.95	112.110	0.29
Emergency Management Directors	1.95	84.310	0.32
Miscellaneous Postsecondary Teachers	1.94	77.650	0.3

Table 2: Ten Lowest Fitness Jobs. They show minimal skill requirements and relatively lower complexities in their roles. Occupations in this list typically involve more routine or physically demanding tasks, with lower average annual wages.

**Job**	**Fitness**	**Average Annual Wages ($)**	**AC**
Graders and Sorters, Agricultural Products	0.1	29.620	0.75
Building Cleaning Workers	0.13	30.490	0.84
Postal Service Workers	0.14	52.500	0.64
Sewing Machine Operators	0.15	29.420	1.0
Dining Room and Cafeteria Attendants and Bartender Helpers	0.15	26.300	0.89
Models, Demonstrators and Product Promoters	0.16	37.630	0.52
Parking Attendants	0.18	27.910	0.55
Billing and Posting Clerks	0.2	41.610	0.63
Mail Clerks and Mail Machine Operators, Except Postal Service	0.21	33.700	0.54
Cutting Workers	0.21	37.690	1.0

3 Heatmaps

3.1 Scatter Plots ($\sigma = 0$)

The heatmaps shown in the main text (Figs. 6 and 7) are obtained by smoothing with a kernel regression the original scatter plots (respectively, left and right panel of Fig. 5 of the main text). The parameter σ of the Gaussian kernel modulates the smoothing: higher values of σ will lead to more uniform heatmaps.

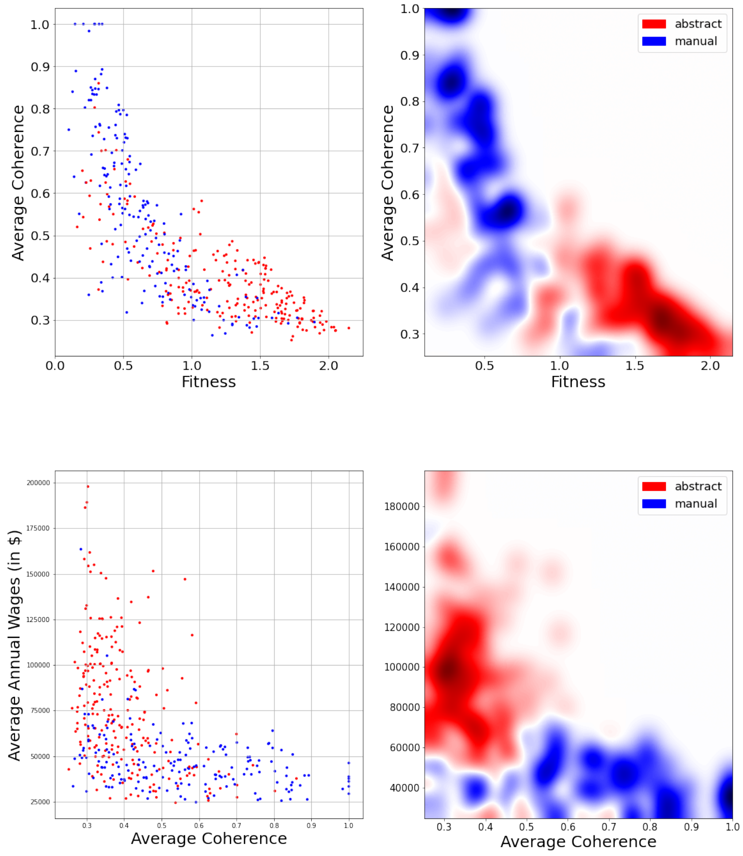

Figure 2: The scatter plots and the smoothing obtained with $\sigma = 32$ related to Fig.6 in the main text.

3.2 Heatmaps at lower σ

In order to stress the non-trivial relation between our measures of job fitness and coherence and the binary classifications already known in the literature, we show in 4 the heatmaps of fig. 6 with a lower value of σ. In this way, the heterogeneity of the original scatter plots emerges, and also the advantage of having continuous instead of binary variables.

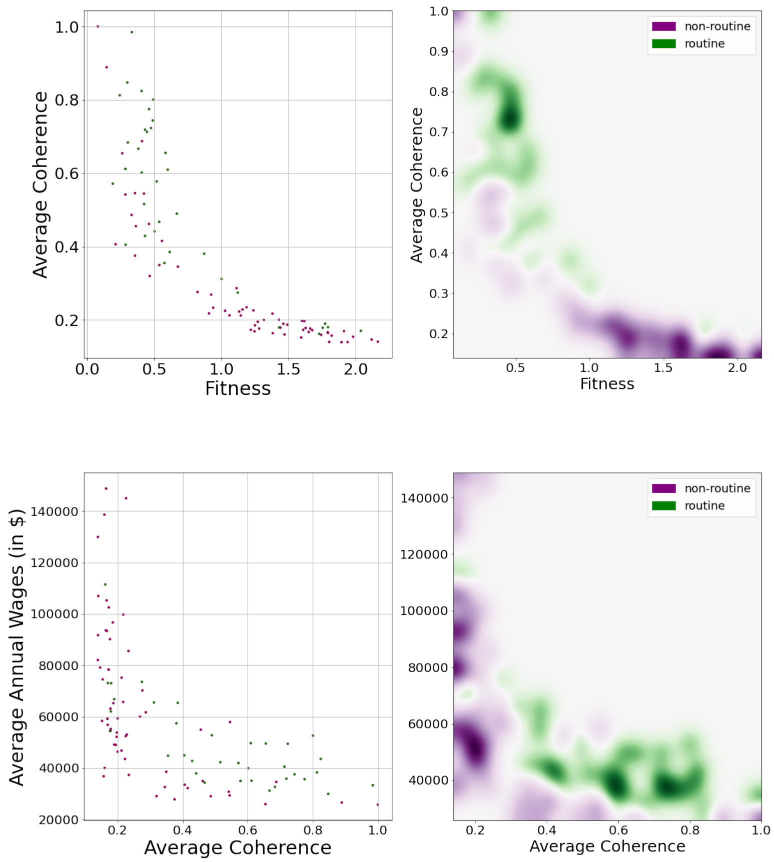

Figure 3: The scatter plots and the smoothing obtained with $\sigma = 32$ related to Fig.7 in the main text.

4 Nestedness of the jobs-skills matrix

In fig. 5 we show a graphical representation of the adjacency matrix $\mathbf{M}$ relative to the bipartite skill-job network. A relatively nested pattern emerges; however: i) Rows and columns are ordered using the Fitness algorithm [**?**], which is known to enhance the visual triangularity of the matrix [**?**], and ii) the possible nestedness of the matrix should be tested against a null model, but the statistical significance strongly depends on the null hypothesis [**?**, **?**]. We thus decided to leave this investigation for future work.

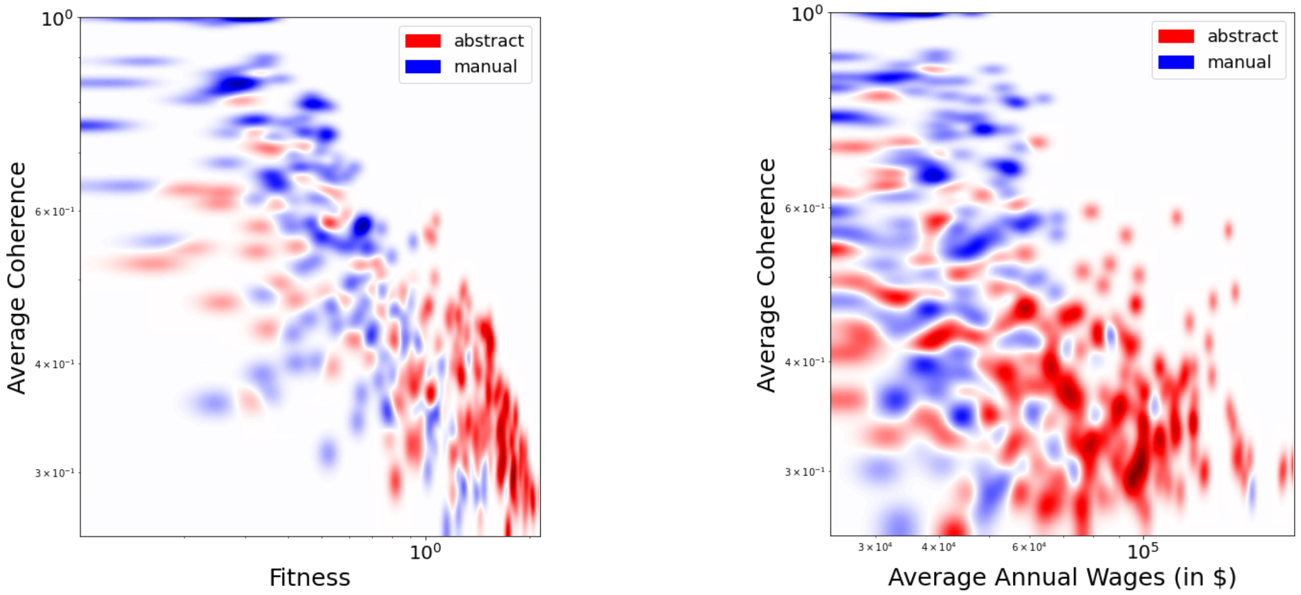

Figure 4: Here we reproduce the heatmaps of figure 6 of the main text, but with a lower smoothing parameter. While the distinction between abstract and manual occupations is robust on average, significant deviations emerge.

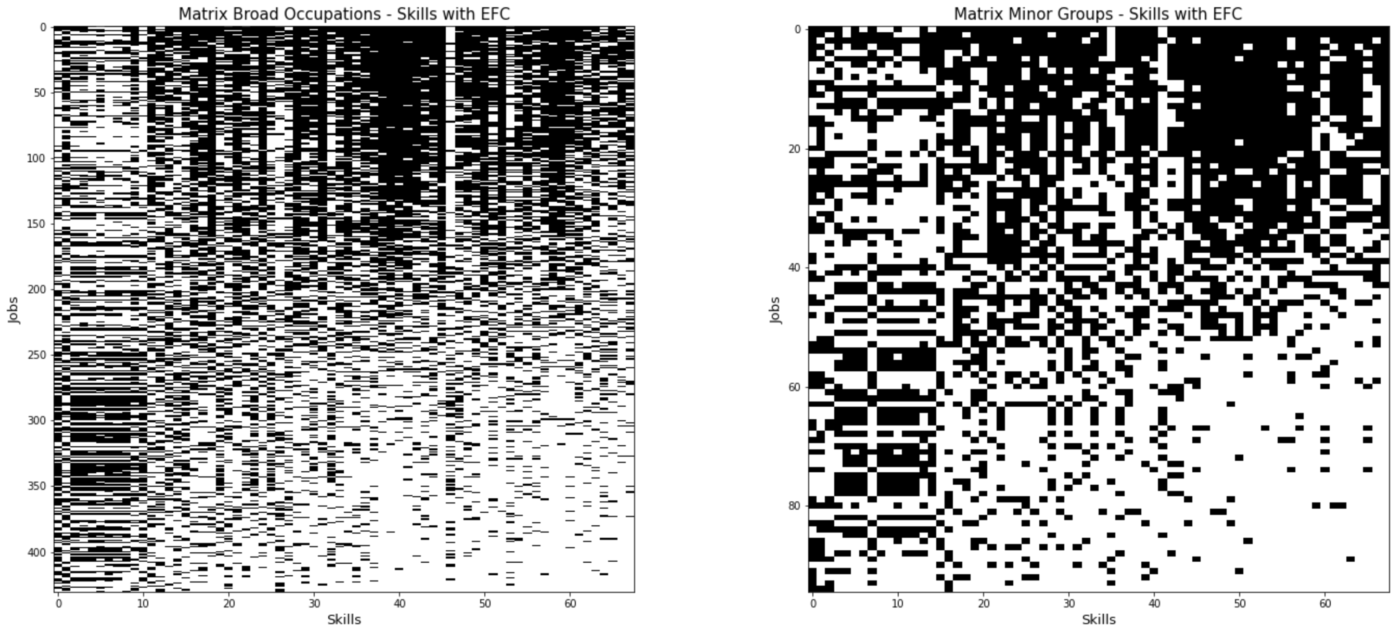

Figure 5: The adjacency matrix of the job-skill network for different job classifications. In both cases, a moderate nested pattern emerges.
